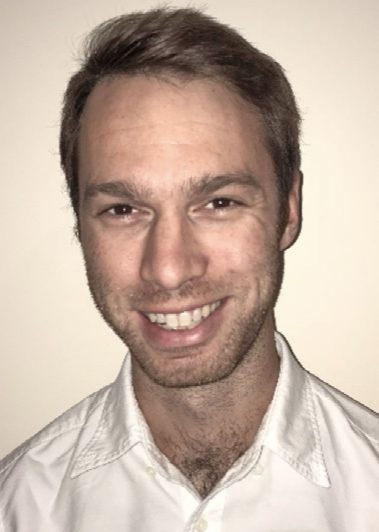# Photochemical Methods for Peptide Macrocyclisation

**DOI:** 10.1002/chem.202003779

**Published:** 2020-10-27

**Authors:** Laetitia Raynal, Nicholas C. Rose, James R. Donald, Christopher D. Spicer

**Affiliations:** ^1^ Department of Chemistry University of York Heslington York YO10 5DD UK; ^2^ York Biomedical Research Institute University of York Heslington York YO10 5DD UK

**Keywords:** macrocyclisation, peptides, photochemistry

## Abstract

Photochemical reactions have been the subject of renewed interest over the last two decades, leading to the development of many new, diverse and powerful chemical transformations. More recently, these developments have been expanded to enable the photochemical macrocyclisation of peptides and small proteins. These constructs benefit from increased stability, structural rigidity and biological potency over their linear counterparts, providing opportunities for improved therapeutic agents. In this review, an overview of both the established and emerging methods for photochemical peptide macrocyclisation is presented, highlighting both the limitations and opportunities for further innovation in the field.

## Graphical abstract

**Recent developments** have allowed photochemical reactions to move to the forefront of the search for new strategies for peptide macrocyclisation. Here, these developments and emerging photochemistries are reviewed, highlighting the opportunities to further innovate in this rapidly expanding field.

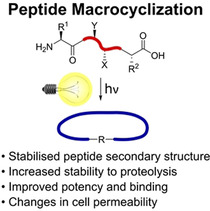

## Introduction

Peptides and small proteins (collectively, PSPs) represent an exciting and emerging frontier in drug discovery, combining the advantages of modularity and synthetic flexibility of small molecules (<500 Da) with the exquisite levels of selectivity and potency of biologics (>5000 Da).[^1^, ^2^, ^3^] PSPs are particularly prized for their capacity to engage in potent and selective interactions with protein surfaces, and hence their ability to modulate protein–protein interactions (PPIs)—therapeutic targets that were once considered “undruggable” using small molecules.[^4^, ^5^] The peptidic nature of PSPs provides them with beneficial attributes such as high binding affinity, biocompatibility and ease and modularity of synthesis, but it is also their Achilles heel. Low cell permeability, poor metabolic stability and unstable secondary structure formation as a result of conformational flexibility, all hinder clinical applications of PSPs.[^6^, ^7^, ^8^, ^9^]

**Figure 1:**
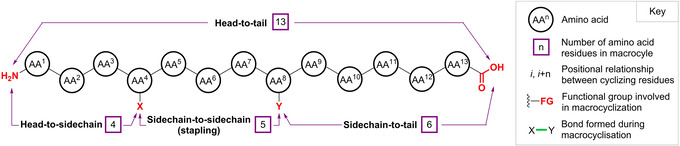
Peptide macrocyclisation modes and generalised layout of figures in this review.

To overcome these limitations, the design and synthesis of macrocyclic PSPs has become increasingly prominent.[^1^, ^2^, ^3^, ^4^, ^8^, ^10^] Macrocyclisation (defined as cyclisation to form rings of ≥12 atoms) can occur through head‐to‐tail, head‐to‐sidechain, sidechain‐to‐tail or sidechain‐to‐sidechain coupling (stapling) (Figure 1), delivering cyclical products, which possess a number of advantageous characteristics over their linear counterparts:^[11]^ i) structures are rigidified, stabilising or enforcing peptide conformations that mimic elements of protein secondary structure^[12]^, α‐helices,[^13^, ^14^, ^15^, ^16^, ^17^, ^18^, ^19^, ^20^] β‐sheets^[21]^ and β‐hairpin turns^[22]^, which would otherwise be unstable can all be induced by cyclisation; ii) cyclised peptides exhibit increased stability to proteolysis, thus prolonging their biological activity and improving their pharmacokinetics. This stability can result from a number of factors, including the poor fit of macrocycles into the active sites of endopeptidases,^[23]^ resistance to the activity of exoproteases that preferentially cleave near the peptide N‐ or C‐termini,^[11]^ or the formation of α‐helices that are resistant to proteolysis due to the presence of a rigidifying, intramolecular, hydrogen‐bonding network;^[17]^ iii) binding efficiency for a target is often improved, an effect classically attributed to cyclic structures being held in conformations better disposed towards binding, with a resultant reduction in the entropic penalty to binding.[^23^, ^24^] However, studies such as those by the groups of Martin and Spaller illustrate a much more complex picture, where pre‐organisation through macrocyclisation may instead strengthen the enthalpic component of binding at the expense of entropy, highlighting the importance of treating macrocycle binding thermodynamics on a case by case basis;[^25^, ^26^, ^27^, ^28^] and iv) cell membrane permeability may be improved, as sidechains can be oriented around the macrocycle in a manner that shields polar atoms from the solvent medium, reducing the polar surface area of the peptide.^[10]^

Given the benefits of PSP macrocyclisation, it is unsurprising that many such compounds occur naturally. A variety of macrocyclic linkages have been identified in natural products, including head‐to‐tail amide bonds, disulfide bridges, biaryls and biaryl ethers.[^29^, ^30^, ^31^, ^32^, ^33^] Chemists have subsequently followed suit, utilising analogous tactics to form cyclic PSPs, as well as developing a diverse toolkit of novel synthetic strategies for macrocyclisation, including ring‐closing metathesis,^[34]^ azide‐alkyne cycloadditions,[^35^, ^36^] other transition‐metal‐catalysed methods,^[37]^ conjugate additions,^[38]^ nucleophilic aromatic substitutions^[39]^ and multicomponent reactions.[^40^, ^41^] These and other methodologies have been extensively reviewed.[^3^, ^18^, ^19^, ^23^, ^37^, ^42^, ^43^, ^44^, ^45^, ^46^, ^47^] Although the coupling chemistry of each of these strategies may differ greatly, common to all of these approaches are a set of challenges that must be overcome, chiefly coaxing the ground‐state *trans* geometries of multiple amide bonds along the PSP backbone into a suitable conformation for cyclisation,^[48]^ and outcompeting deleterious oligomerisation reactions.

In contrast to the methods outlined above, the use of photochemical methods for PSP cyclisation had until recently been relatively underexplored. However, in line with a wider renaissance of the fields of photo‐ and radical chemistry, recent developments have brought this area to the fore. Photochemical strategies are highly attractive for macrocyclisation: reactive open‐shell or photoexcited‐state species are known to readily participate in macrocyclisation through mechanisms that are fundamentally different to their two‐electron ground‐state counterparts, offering ample scope for novel cyclisation manifolds that overcome some of the limitations of two‐electron and transition‐metal‐catalysed methods.[^49^, ^50^] Given that most peptides are largely transparent to light of wavelengths >320 nm (and those without aromatic amino acids or disulfide bonds, >250 nm),^[51]^ photochemical methods allow targeted excitation of chromophores in a reaction mixture, affording the potential for both mild and highly selective processes to take place, which are compatible with complex biological systems.[^52^, ^53^, ^54^] Furthermore, the precise control of photochemical processes afforded by manipulating the incident light, both spatially and temporally, offers much scope for applications of these emerging methods in biomedical settings. Herein, we therefore review the application of light‐driven processes for PSP macrocyclisation for the first time. It is our hope that through this summary of the field we will promote increased uptake of these powerful methodologies, and help stimulate further development in this exciting and rapidly evolving area.

## Redox‐Neutral Photochemical Peptide Macrocyclisation

### Introduction

Photochemical macrocyclisation reactions can typically be grouped into two categories: i) redox‐neutral processes where the photoexcited state either directly takes part in the macrocyclisation, or produces reactive species through atom transfer or bond rearrangement, which then participate in cyclisation; and ii) those where the photoexcited state first engages in photoinduced electron transfer (PET) to provide radical, radical ion or organometallic species, which are necessary for cyclisation. In this section, we will focus on the first class of reactions, where photochemical macrocyclisation can be further categorised based on the specific behaviour of the photoexcited state: hydrogen atom transfer (HAT); bond reorganisation; or cycloaddition. Photoactivatable motifs are widespread in the bioconjugation field, however, in this review we will focus only on reactions with levels of chemoselectivity that allow their use for controlled cyclisations. Thus, widely used reactive handles in photoaffinity labelling, such as diazirines and aryl azides, which generate highly reactive species upon activation that can react in a promiscuous, largely non‐selective manner, will not be discussed.

### Macrocyclisation triggered by photoinitiated hydrogen atom transfer (HAT)

#### Thiol‐“ene” reactions

Thiols represent a versatile reactive handle for peptide and protein modification that can undergo a wide range of different chemistries. As a result, the modification of cysteine residues has been widely used for peptide macrocyclisation via alkylation,[^55^, ^56^] arylation^[39]^ and disulfide formation.^[57]^ Conjugate addition reactions,[^38^, ^58^] also referred to as nucleophilic thiol‐“ene” reactions, are particularly popular owing to their chemoselectivity, mild reaction conditions and rapid kinetics.^[59]^ However, the need to use activated, electrophilic alkenes can lead to possible side reactions with other nucleophilic amino acids, particularly lysine.^[60]^ The highly selective reaction of thiyl radicals with unactivated alkenes, via a photo thiol‐“ene” mechanism, is therefore an attractive alternative.^[61]^

**Figure 2:**
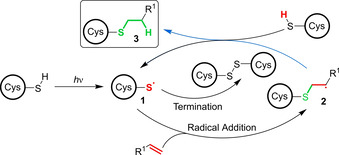
Mechanism of the photoactivated thiol‐“ene” reaction. A photoinitiator can be used to accelerate the formation of a thiyl radical via S−H abstraction.

Thiol radical formation (**1**, Figure 2) can be initiated through direct photolysis of the thiol with UV light. Although thiyl radical generation is slow at wavelengths >280 nm (e.g., when using a sunlamp with pyrex filter), this can be advantageous for chain processes where a low steady‐state concentration of radicals helps suppress deleterious processes.^[61]^ More often, a photoinitiator is employed and thiyl radicals are generated by a rapid HAT between the thiol and either the photoexcited state initiator directly, or daughter radicals stemming from its photo‐decomposition (e.g., for HAT between **^.^**CH_3_ and EtSH, *k*_298K_≈5×10^7^ m ^−1^ s^−1^).^[62]^ Thiyl radical **1** subsequently adds across an alkene C=C bond in an anti‐Markovnikov manner, generating a carbon‐centred radical **2**. Abstraction of a hydrogen atom from another thiol generates the hydrothiolated product **3** and an additional thiyl radical **1**, facilitating chain propagation.

The steric and electronic nature of the alkene substituents play a significant role in governing the efficiency and outcome of this process. A fine balance exists between the forward (and reverse) rates of thiyl radical (**1**) addition to an alkene, S‐H abstraction from another thiol by the resultant carbon‐centred radical **2** and competitive addition of **2** to another alkene to afford off‐cycle oligomerisation/polymerisation products.[^63^, ^64^, ^65^] In general, electron‐rich alkenes typically react more rapidly, with terminal alkenes being similarly more reactive than internal analogues.^[63]^ Norbornene and vinyl ether derivatives undergo exclusively hydrothiolation over oligo‐/polymerisation owing to particularly rapid thiyl radical additions, as a consequence of strain release and polarity matching, respectively, and fast S‐H abstraction steps. Although the competitive formation of disulfide bonds is typically slow, recent reports by the Bowman group have highlighted the potential role of thiolate anions in accelerating this side reaction, through the formation of a metastable disulfide radical anion.^[66]^ However, although this may be problematic in the context of thiol‐“ene” polymerisations, the high effective concentration of alkene during intramolecular macrocyclisation likely negates this effect.

**Figure 3:**
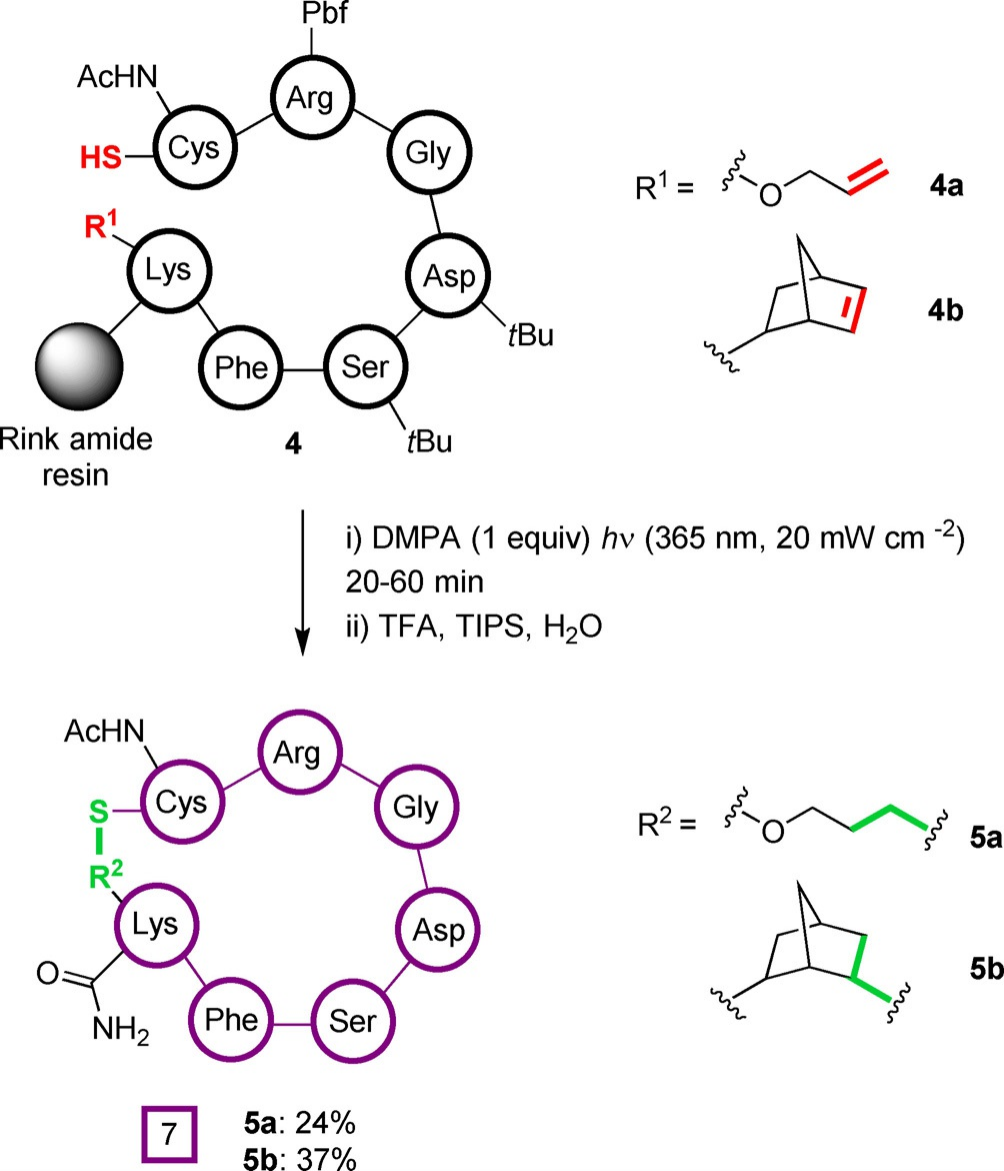
First example of peptide macrocyclisation by a photoactivated thiol‐“ene” reaction. On‐resin cyclisation onto both alloc and norbornene alkene partners was achieved in the presence of a DMPA photoinitiator.^[67]^

In the earliest example of photochemical thiol‐“ene” peptide macrocyclisation, Aimetti et al. demonstrated the on‐resin synthesis of an integrin‐binding cyclic Arg‐Gly‐Asp (RGD) peptide (Figure 3).^[67]^ Irradiation of resin‐bound peptides **4**, containing unprotected cysteine residues, at 365 nm in dimethylformamide (DMF) led to cyclisation with either allyloxycarbonyl (alloc; **4 a**) or amido‐norbornene (**4 b**) modified lysine residues in the presence of the photoinitiator 2,2‐dimethoxy‐2‐phenylacetophenone (DMPA). As expected, given the reactivity profiles of different alkenes discussed above, higher yields and shorter reaction times were reported for **4 b** than **4 a** (37 % vs. 24 %, respectively). Notably, photoactivation was shown to be both quicker and more effective than the use of the thermally activated radical initiator azobisisobutyronitrile (AIBN). As all other amino acids in the peptide sequence were protected, potential side reactions were minimised and the cyclised peptides **5 a** and **5 b** were efficiently obtained following cleavage. Macrocyclisation of the solution‐phase, fully deprotected peptide was also performed in methanol. Although conversions were comparable, the need for subsequent peptide purification led to overall lower yields.

Building upon this work, the same authors demonstrated that peptides could undergo further chain extension following on‐resin thiol‐“ene” macrocyclisation.^[68]^ This allowed the installation of a second reactive cysteine for subsequent conjugation of a cyclic‐RGD motif to a multivalent polymer backbone. Importantly, a combination of cyclic and multivalent peptides was found to act synergistically to enhance inhibition of fibrinogen binding to glycoprotein IIb/IIIa, a key integrin found on the surface of platelets, by up to two orders of magnitude.

**Figure 4:**
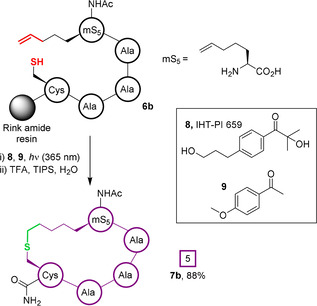
On‐resin macrocyclisation of unactivated alkenes by a photoactivated thiol‐“ene” reaction.^[69]^

The use of unactivated, aliphatic alkenes for on‐resin photo thiol‐“ene” macrocyclisation was subsequently reported by Zhao et al.^[69]^ Peptides containing unnatural amino acids with but‐4‐ene, pent‐5‐ene, or hex‐6‐ene sidechains (**6 a**–**c**, respectively) were cyclised with a cysteine residue at the *i+*4 position under 365 nm irradiation in DMF, with comparable yields (**7 a**–**c**, 79–90 %; Figure 4). Of the radical initiators screened, 2‐hydroxy‐4′‐(2‐hydroxyethoxy)‐2‐methylpropiophenone (**8**, also known as IHT‐PI 659) was found to give the highest conversion in the presence of 4‐methoxyacetophenone (**9**), an additive that was observed to reduce byproduct formation, facilitating product purification by HPLC.

In 2015, Wang et al. reported the photo thiol‐“ene” macrocyclisation of peptides composed solely of natural amino acids through a novel solution‐phase, two‐component approach.^[70]^ A series of bifunctional dienes were used to link two cysteine residues under 365 nm irradiation, in the presence of a DMPA photoinitiator. Although the reaction was compatible with DMF, the highest yields were obtained in *N*‐methyl‐2‐pyrrolidone (NMP). This two‐component approach offers synthetic versatility, enabling the generation of a library of peptides cyclised with different diene linkers from a single peptide precursor. This is particularly important given the strong influence of cross‐linker structure on peptide properties. For example, changes in lipophilicity have been shown to impact the ability of cyclic peptides to cross phospholipid bilayers and therefore the potency of therapeutic peptides,^[71]^ whereas hydrogen‐bonding interactions have been shown to influence peptide conformation and target binding.^[72]^ As a result, the authors were able to develop bis‐thioether cyclised peptide inhibitors of p53‐HDM2 interactions, which were able to induce the apoptosis of colorectal carcinoma cells with a comparable potency to previously reported hydrocarbon‐linked peptides generated by ring‐closing metathesis.^[73]^

**Figure 5:**
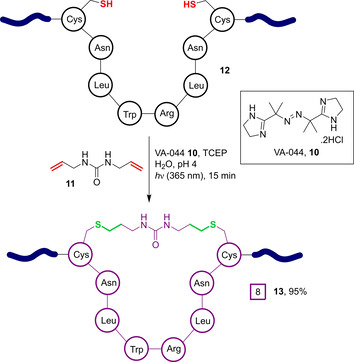
Aqueous photoactivated thiol‐“ene” reaction, enabled by the use of the water‐soluble initiator VA‐044 **10**.^[74]^

More recently, the same authors have developed this two‐component approach further to enable the macrocyclisation to be performed in water.^[74]^ To overcome challenges with aqueous solubility previously encountered by other groups, the water‐soluble photoinitiator 2,2′‐azobis[2‐(2‐imidazolin‐2‐yl)propane]‐dihydrochloride (also known as VA‐044, **10**) was employed to induce thiol conjugation to water‐soluble diallyl‐urea **11** (Figure 5). At pH 4 in aqueous solution, the reducing agent TCEP (tris(2‐carboxyethyl)phosphine) was found to greatly enhance the conversion of peptide **12** to cyclised **13** from 53 % to 95 % by minimising competing disulfide formation.

**Figure 6:**
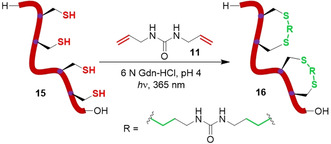
Aqueous di‐cyclisation of a tetra‐cysteine protein mutant by photoinitiated thiol‐“ene” chemistry.^[74]^

Impressively, the macrocyclisation of a 9 kDa, dicysteine, coiled‐coil protein substrate was also demonstrated. Although the addition of TCEP was found to be detrimental in this scenario owing to competitive desulfurisation, in its absence the reaction was found to proceed effectively in mildly acidic (pH 4) acetate buffer containing denaturing guanidine hydrochloride (Gdn‐HCl). This reduced disulfide formation and maximised cyclisation efficiency, respectively, leading to 90 % conversion to stapled protein **14**. Double cyclisation of a tetra‐cysteine mutant **15**, containing two separate *i*,*i+*7 cysteine pairs to generate **16**, was also found to proceed with 80 % conversion (Figure 6). Although not reported by the authors, the presence of undesired linkages between the two separate cysteine pairs cannot be ruled out. However, it is likely that the spatial proximity of the *i*,*i+*7 residues would strongly favour cyclisation between adjacent cysteines, even under denaturing conditions.

#### Thiol‐“yne” reactions

**Figure 7:**
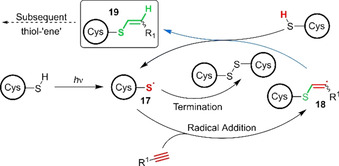
Mechanism of the photoactivated thiol‐“yne” reaction. A photoinitiator can be used to accelerate the formation of a thiyl radical by S−H abstraction.

In an analogous fashion to thiol‐“ene” reactions, thiols can also react with alkynes by both nucleophilic and radical mechanisms. Photoinitiated thiol‐“yne” reactions with unactivated alkynes therefore offer an alternative strategy for achieving PSP macrocyclisation. Conjugation proceeds through a mechanism similar to that for thiol‐“ene” reactions, comprising light‐initiated formation of thiyl radical **17**, addition to an alkyne to form **18** in an anti‐Markovnikov manner and subsequent hydrogen atom abstraction by vinyl radical **18** (Figure 7). However, the thiol‐“yne” reaction differs in that it generates a vinyl sulfide product **19**, which is itself susceptible to further modification in the presence of excess thiol, through a subsequent thiol‐“ene” reaction. Although less widely used than the analogous “ene” reaction, thiol‐“yne” conjugations therefore offer intriguing possibilities for achieving double modification or dual functionality,^[75]^ although these properties have yet to be exploited by the PSP macrocyclisation community. Indeed, to date there has only been a single report of photoactivated thiol‐“yne” mediated peptide macrocyclisation.

**Figure 8:**
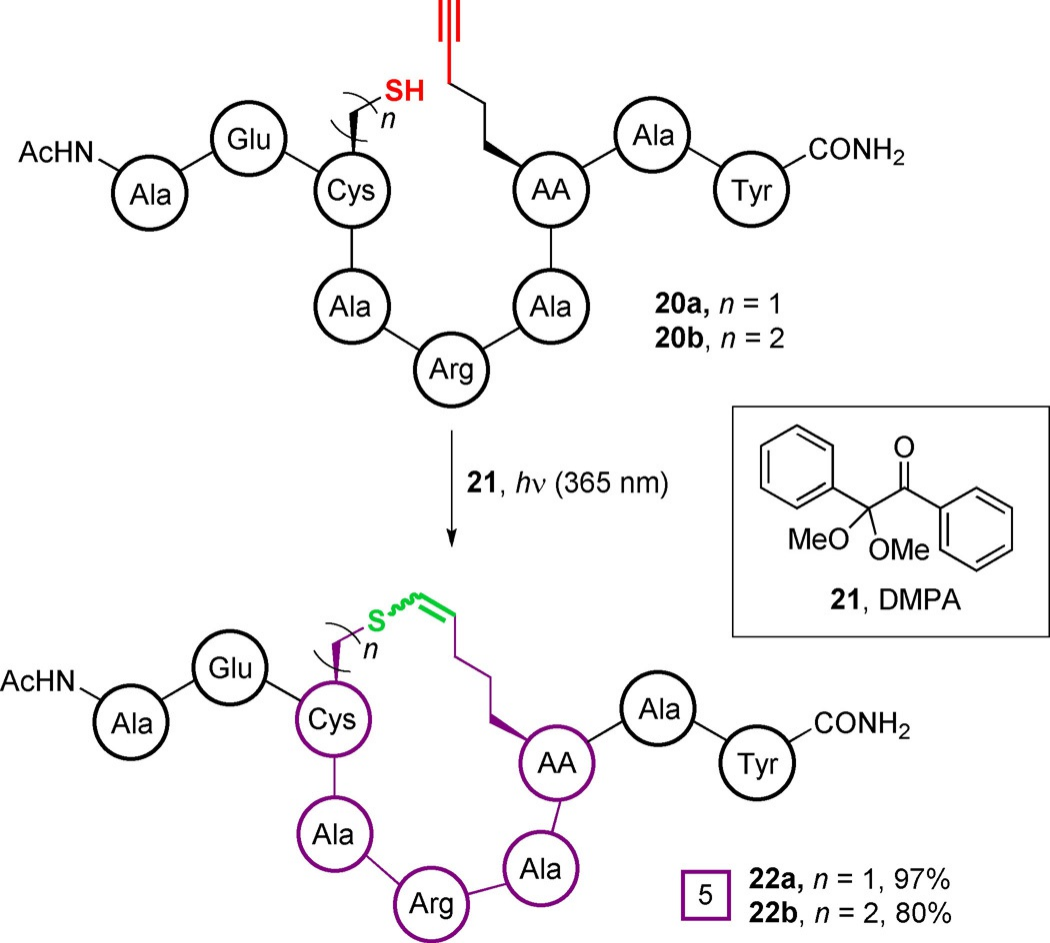
Photoactivated thiol‐“yne” macrocyclisation to generate a vinyl sulfide‐linked cyclic peptide.^[71]^

Tian et al. demonstrated the intramolecular photo thiol‐“yne” cyclisation of peptides containing an unnatural amino acid bearing a pent‐5‐yne sidechain, and either cysteine (**20 a**) or homocysteine (**20 b**) at the *i+*4 position (Figure 8).^[71]^ A screen of photoinitiators found IHT‐PI 659 (**8**) and 2,2‐dimethoxy‐2‐phenylacetophenone (DMPA, **21**) to provide the highest conversions, with a strong preference for the formation of the *Z*‐vinyl sulfide products **22 a** and **22 b**. The resultant peptides were designed to modulate intracellular interactions between the oestrogen receptor and its coactivators, a key target for the treatment of certain cancers and osteoporosis. Importantly, the vinyl sulfide‐containing macrocycle generated was proposed to provide increased rigidity, and to enhance the α‐helical character over the alkyl sulfide linker, which would be generated by an analogous thiol‐“ene” cyclisation. Furthermore, comparison to an all hydrocarbon linked analogue generated by ring‐closing metathesis indicated that the vinyl sulfide contributed to greatly reduced membrane toxicity. Thus, this report highlights the importance of the cyclisation linker structure in determining the properties, both physical and biological, of a cyclised peptide. Rather than being interchangeable with the thiol‐“ene” reaction, photo thiol‐“yne” reactions should be considered an important addition to the macrocyclisation toolbox that can generate a distinct vinyl sulfide linkage, which may possess unique properties.

#### Benzophenone‐methionine conjugation

**Figure 9:**
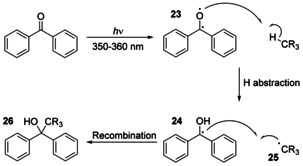
Mechanism of the intermolecular reaction between a photoexcited benzophenone and an accessible C−H bond.

The ability of certain ketones and aldehydes to form reactive species under UV irradiation has been widely exploited in organic synthesis and chemical biology. Benzophenones undergo photoexcitation on irradiation with longer wavelength UV light (350–360 nm),[^76^, ^77^] and importantly, relative to other photoactivatable groups such as diazirines and aryl azides, form intermediates that exhibit useful (albeit limited) levels of chemoselectivity. Mechanistically, the highly reactive diradical **23** generated following irradiation can abstract a hydrogen atom from an accessible C−H bond to generate an α‐hydroxy radical **24** and carbon‐centred radical **25** (Figure 9). Pairing of these two species leads to the formation of a new carbon–carbon bond in tertiary alcohol **26**.

In an attempt to understand and harness preferential reactivity, Deseke et al. studied the regio‐ and chemoselective C−H abstraction of irradiated benzophenones with a panel of *N*‐acetyl amino acid methyl esters.^[78]^ In acetonitrile, the highest reactivity was observed with glycine owing to its readiness to undergo hydrogen atom abstraction from the α‐carbon (51 % conversion). Methionine was also found to be preferentially modified (40 % conversion), with alkylation occurring either at the γ‐ or ϵ‐carbon atoms adjacent to sulfur. Conversely, in a 4:1 mixture of pyridine/water, methionine modification was found to be favoured (45 % conversion), with a corresponding drop in glycine modification (16 %). The authors proposed that this change in selectivity was due to competitive base‐catalysed degradation of adducts formed at the α‐position of glycine. Although modification of these two amino acids was dominant, low levels of reactivity with several other amino acids was also observed. Indeed, only residues containing primary amides or carboxylic acids (aspartic acid, asparagine, glutamic acid and glutamine) were found to be inert.

**Figure 10:**
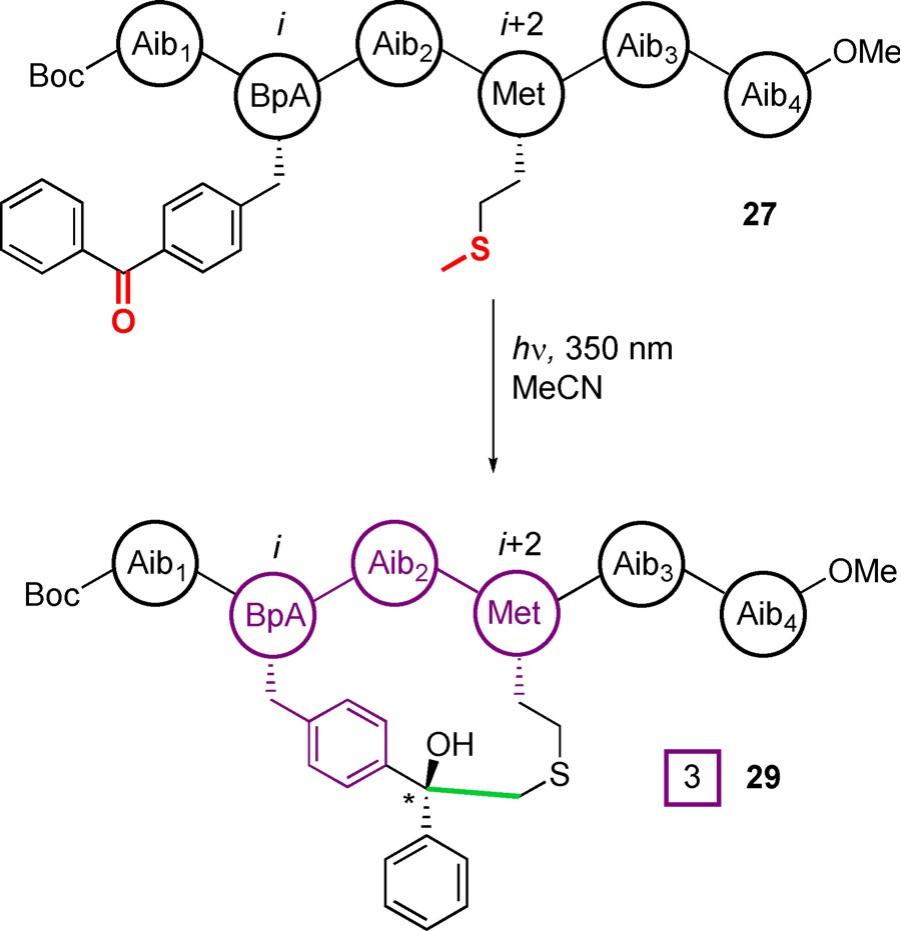
Macrocyclisation through the conjugation of benzophenone to methionine under UV irradiation. By placing the unnatural amino acid Aib at all other positions, potential side reactions with α‐hydrogens are prevented.^[80]^

Building on this preferential reactivity at glycine and methionine, Moretto et al. reported the first use of benzophenone photoactivation for intramolecular peptide cyclisation in 2009.[^79^, ^80^] Hexapeptides **27** containing the unnatural benzophenone‐based amino acid Bpa (**28**) and methionine were synthesised, and their positions in the peptide chain varied to study the effects of distance on macrocyclisation efficiency (Figure 10). To prevent unwanted reactions at other sites in the peptide, the di‐α‐substituted unnatural amino acid 2‐aminoisobutyric acid (Aib, or α‐methylalanine) was installed at all other positions owing to its known inertness to C−H abstraction, and ability to promote α‐helix formation. Cyclisation was successful when methionine was placed at the *i+*1, *i+*2, *i+*3, or *i+*4 positions relative to Bpa, generating cyclised peptides **29**. For an analogous nonapeptide **30**, cyclisation to the *i*−3 and *i+*3 positions was found to occur exclusively at the ϵ‐carbon of methionine, with the two diastereomers generated by the new chiral tertiary alcohol being formed in an approximately 1:1 ratio.

**Figure 11:**
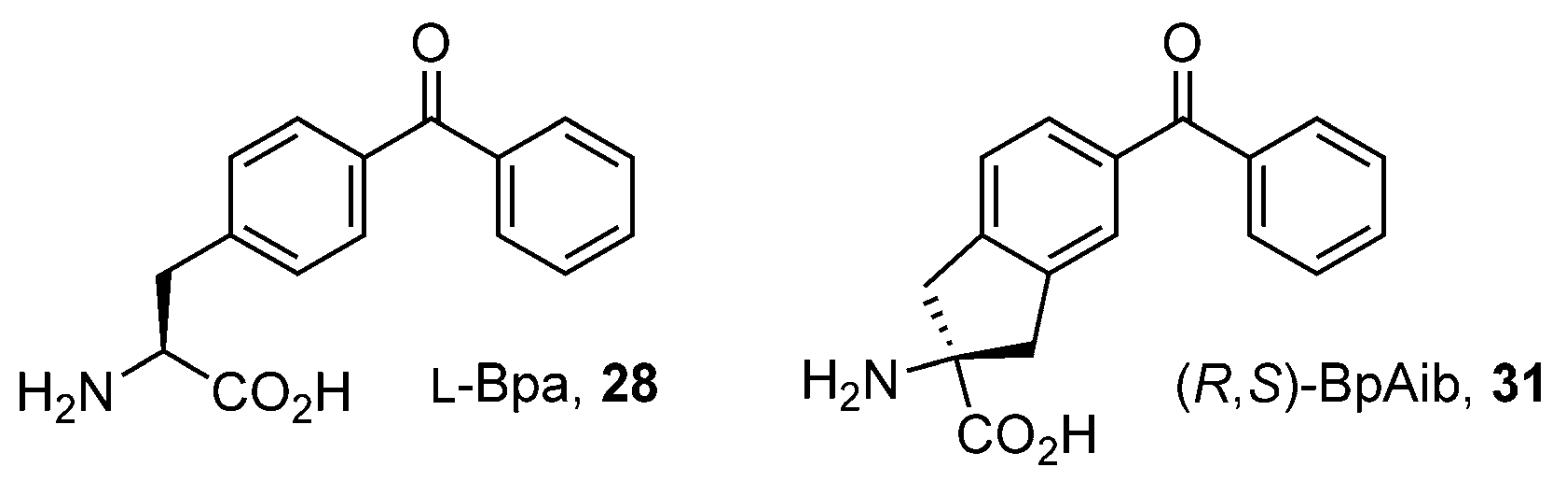
Structures of the unnatural benzophenone‐containing amino acids Bpa **28** and BpAib **31**.

Building upon this work, Wright et al. subsequently introduced a new α‐C‐tetrasubstituted, cyclic, benzophenone‐based amino acid BpAib (**31**), with increased structural rigidity (Figure 11).^[81]^ Although the photoactivated macrocyclisation of this amino acid with methionine was demonstrated, efforts to exploit the benefits of decreased conformational freedom imparted by BpAib have yet to be reported.

Lewandowska‐Andralojc et al. have subsequently demonstrated that Bpa‐methionine cyclisation can take place between the two sidechains of a cyclic dipeptide to form a rigid bridged macrocycle.^[82]^ This study highlighted that the C−H abstraction step to form an α‐hydroxy radical is in fact reversible. Furthermore, C−C bond formation was found to take place selectively at the methionine δ‐carbon to minimise the significant ring strain imposed by cyclisation. Thus, both steric and chemical factors are at play in dictating C−H selectivity.

The ability of benzophenones to cyclise with natural amino acids following irradiation is an advantage to their use. However, the lack of specificity resulting from off‐target reactions with alternative amino acids, particularly glycine, is severely restricting. This is highlighted by the limited number of reports of benzophenone PSP macrocyclisation, and the even more striking lack of diversity in the amino acids that have been integrated into the peptide substrates. Moreover, the formation of diastereomeric products, resulting from the creation of a new chiral centre, may be problematic, potentially requiring separation and complex purification to generate a homogeneous cyclised product. As such, the photoactivation of benzophenones as a means to control PSP macrocyclisation is unlikely to find increasing prominence in the coming years, given the advantages of many of the other reactions presented in this review.

### Photoactivation via bond reorganisation

#### Photorearrangement of 7‐nitroindoline amides

**Figure 12:**
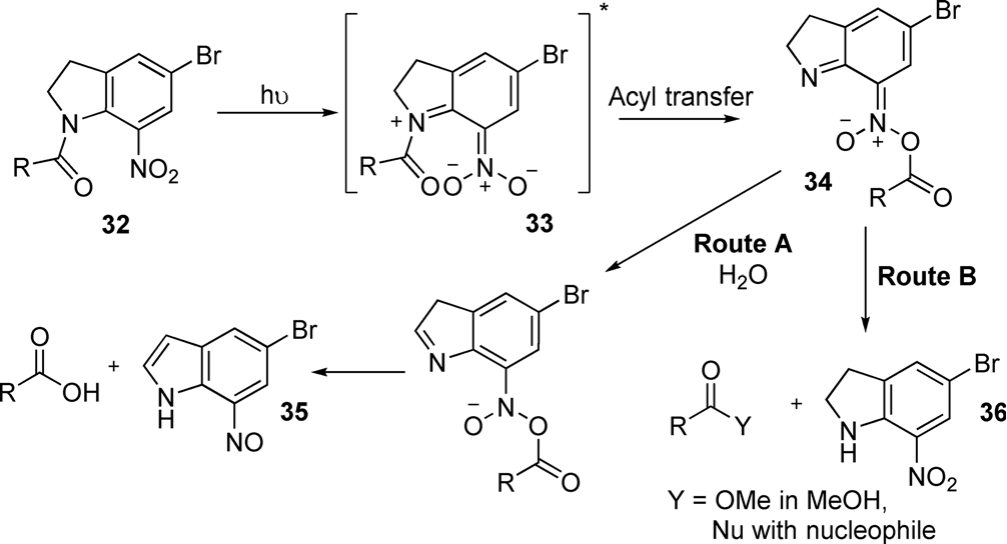
Mechanism of 5‐bromo‐7‐nitroindoline (Bni)‐amide photoactivation and nucleophilic cleavage. In water, intermediate **34** generates nitroso‐indole **35**. In contrast, in the presence of a nucleophile or nucleophilic solvent nitro‐indoline **36** is generated.[^83^, ^84^]

In 1999, Papageorgiou and co‐workers proposed a mechanism for the solvolysis of 5‐bromo‐7‐nitroindoline (Bni)‐amides (**32**), and related 5‐substituted derivatives, following photoactivation.[^83^, ^84^] Irradiation of **32** with UV light generates a photoexcited state **33**, which undergoes N→O acyl transfer to generate the highly activated intermediate **34** (Figure 12). In water, **34** undergoes cleavage to generate 7‐nitroso‐indole **35** with the release of a free carboxylic acid. However, in the presence of a competing nucleophile or nucleophilic solvent, attack at the electrophilic carbonyl leads to cleavage of the Bni motif with the release of 7‐nitro‐indoline **36**.

The propensity to undergo hydrolysis under irradiation makes Bni‐derivatives useful protecting groups, but also severely impacts their bioconjugation efficiency in aqueous media. However, in non‐nucleophilic organic solvents, nucleophilic attack by amines can be used to induce amide formation following photoactivation. This chemistry has therefore been exploited for intramolecular photocyclisation and in particular as a useful method for macrolactamisation.^[85]^ Indeed, compared with other macrolactamisation strategies that require the use of added coupling agents, the irradiation of Bni‐amides provides a facile means to induce cyclisation without the need for additives.

**Figure 13:**
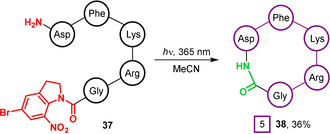
Solution‐phase head‐to‐tail macrocyclisation of a peptide containing a C‐terminal Bni‐amide under UV irradiation.^[86]^

The capacity of photoactivated Bni‐amides to react with nucleophiles was exploited by Mifune et al. for PSP macrocyclisation.^[86]^ This approach is attractive as it generates a native peptide bond and the resultant macrocycles are therefore able to mimic natural cyclic peptides. Indeed, the authors demonstrated that a synthetic pentapeptide **37**, bearing a C‐terminal Bni amide, could be cyclised in a 36 % yield following irradiation at 365 nm under flow conditions, generating a previously reported cyclic RGD sequence **38** in situ that can act as a selective antagonist of the α_v_β_3_ integrin receptor (Figure 13).[^87^, ^88^, ^89^, ^90^, ^91^] The absence of excess activating agents or catalysts during cyclisation greatly facilitated purification of the cyclised peptide, and the ability of the Bni group to play a parallel role as a C‐terminal protecting group during peptide synthesis was also highlighted by the authors as a notable advantage. However, the presence of a C‐terminal Bni‐amide also necessitated the use of solution‐phase peptide synthesis. Although this was achieved by using micro‐flow peptide synthesis, such technologies are not as widespread as solid‐phase approaches and the generality and translatability of this approach may therefore be limited at present.^[92]^ Post‐synthesis derivatisation is another possible route to achieve C‐terminal Bni‐amide installation for head‐to‐tail or sidechain‐to‐tail cyclisations but also presents synthetic challenges. In contrast, head‐to‐sidechain or sidechain‐to‐sidechain couplings would not experience these difficulties and the installation of a Bni‐motif could plausibly be achieved in a straightforward manner on‐resin. However, the need to also protect Asp, Lys and Arg residues to prevent side reactions remains a significant limitation of this strategy, and as a result there remains only a single report of Bni photoactivation in the peptide macrocyclisation literature to date.

#### Photorearrangement of tetrazoles

**Figure 14:**
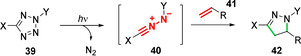
Mechanism of 2,5‐diaryl tetrazole photoactivation to form a nitrile imine, and subsequent 1,3‐dipolar cycloaddition with a suitable dipolarophile.

Building on the pioneering work of Huisgen, in 2007, the Lin group reported the use of 2,5‐diaryl tetrazoles as photoactivatable precursors for 1,3‐dipolar cycloadditions.^[93]^ As these reactions are greatly accelerated in aqueous media, they have emerged as useful tools for photoactivated bioconjugation. Upon UV irradiation of tetrazole **39**, expulsion of nitrogen gas generates a highly reactive nitrile imine **40**, which can serve as a 1,3‐dipole for cycloaddition with a suitable dipolarophile **41**, most commonly an alkene, to afford a pyrazoline cycloadduct **42** (Figure 14). Cycloaddition proceeds with high regioselectivity, particularly with electron‐deficient alkenes, generating **42** as a single regioisomer.^[93]^ When photolysis is rapid, cycloaddition becomes rate‐determining and reactions follow apparent second‐order kinetics.

The chemical versatility of the 2,5‐diaryl scaffold is attractive as it provides significant scope to tune the properties of the tetrazole and intermediate nitrile imine. For example, by variation of the aryl substituents X and Y, the wavelength sensitivity can be tuned within the UV region.^[94]^ Similarly, the rate of cycloaddition can be increased by selecting substituents that serve to raise the highest occupied molecular orbital (HOMO) energy of nitrile imine **40**.^[95]^ Monoaryl tetrazoles are also able to eliminate nitrogen under irradiation and can undergo efficient cycloaddition, albeit at a significantly reduced rate.^[96]^

**Figure 15:**
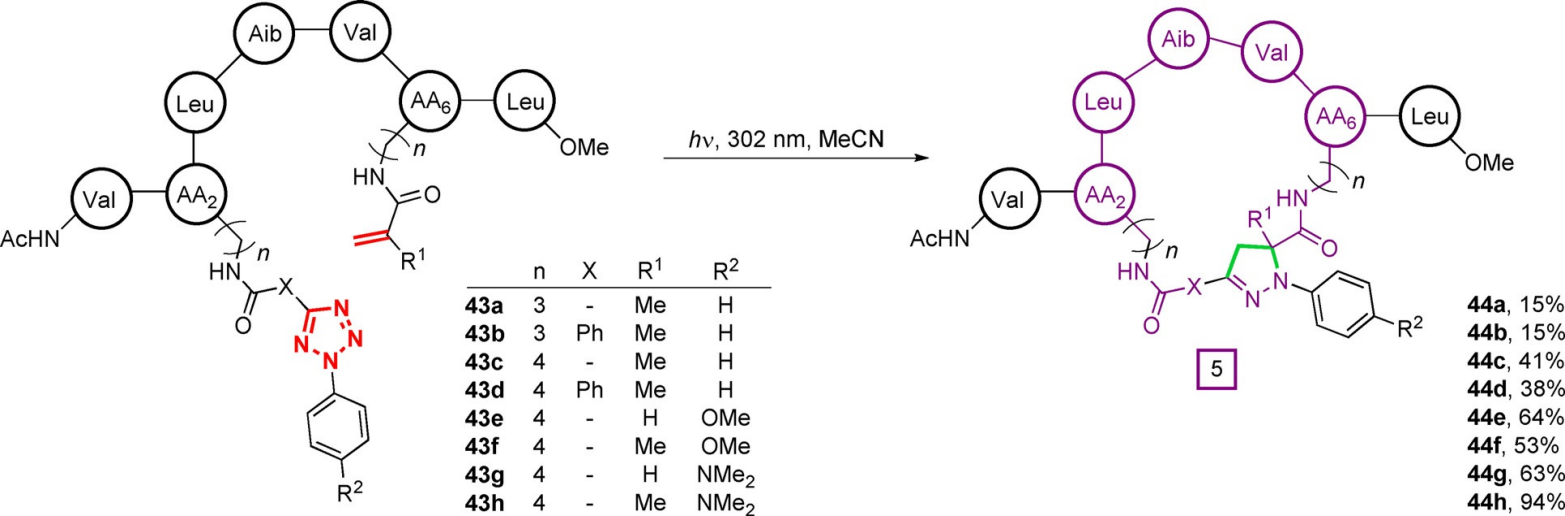
Macrocyclisation of tetrazole‐ and alkene‐containing peptides under UV irradiation, by the formation of an intermediate reactive nitrile imine and subsequent 1,3‐dipolar cycloaddition.^[97]^

The first use of this chemistry for peptide macrocyclisation was reported by Madden et al. in 2009.[^97^, ^98^] Tetrazole‐ and (meth)acrylamide‐based unnatural amino acids were introduced into a synthetic heptapeptide **43** and irradiated at 302 nm, to trigger nitrile imine formation and subsequent 1,3‐dipolar cycloaddition to form **44** (Figure 15). Nitrile imines generated from both mono‐ and di‐aryl tetrazoles were able to undergo macrocyclisation with comparable efficiency, although sidechain flexibility was found to be a major determinant of cyclisation efficiency. Functionalised amino acids based on a shorter chain ornithine core (**43 a**,**b**, *n*=3, 15 %) underwent cyclisation with lower efficiency than those based on lysine (**43 c**–**h**, *n*=4, 40 %). Similarly, macrocyclisation efficiency was found to depend on the structure of both the tetrazole and alkene reactive partners. Electron‐donating substituents on the aryl tetrazole (R^2^=OMe, **43 e**,**f**) greatly increased the conversion by increasing the HOMO energy of the intermediate nitrile imine, therefore accelerating cycloaddition. Interestingly, methacrylamide derivatives (R^1^=Me, **43 f**,**h**) were found to react more efficiently than the corresponding acrylamides (R^1^=H, **43 e**,**g**) despite previous small molecule studies to the contrary.^[94]^ Acrylamides are known to possess increased reactivity towards dipolar cycloaddition, suggesting that conformational freedom is likely to play a significant role in dictating peptide cyclisation efficiency. Interestingly, the conversion of monoaryl or diaryl tetrazoles was found to be comparable despite the lower reactivity of monoaryl derivatives, again suggesting a conformational influence on cyclisation.^[96]^

The high selectivity reported for nitrile imine‐alkene cycloadditions would appear to make 2,5‐di‐aryltetrazoles interesting motifs for light‐induced peptide macrocyclisation. However, it is important to note that side reactions of the highly reactive nitrile imine with natural amino acid sidechains have been reported.^[99]^ This may limit macrocyclisation efficiencies in systems where the tetrazole and alkene reactive pair are unfavourably positioned, but does open up the intriguing possibility of using tetrazoles as light‐activatable reagents for cyclisation with proteinogenic amino acids.

### Photoinitiated cycloaddition

**Figure 16:**
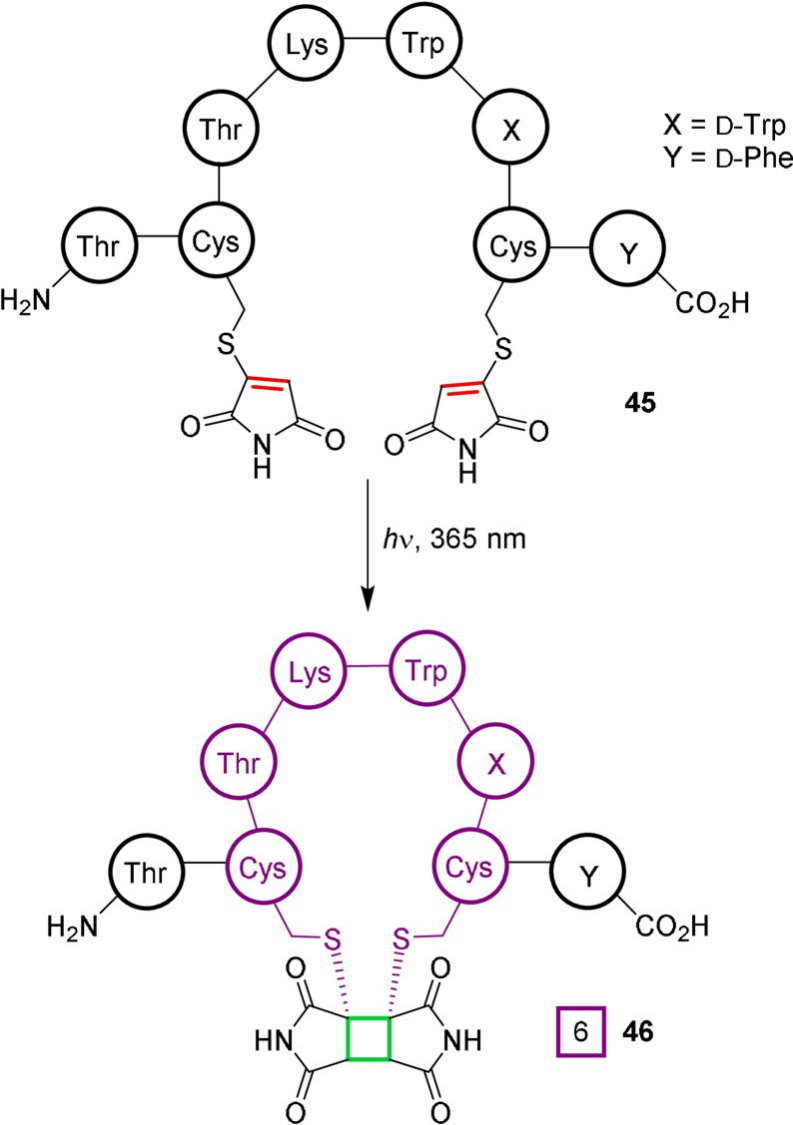
Thiomaleimide [2+2] cycloaddition under UV irradiation to generate a macrocyclic peptide.^[101]^

In the previous section, photoactivation led to the generation of a reactive intermediate that could subsequently undergo cycloaddition. An alternative strategy is to exploit photoexcited‐state molecules that can themselves directly undergo cycloaddition. For example, following photoexcitation with UV light, maleimide molecules readily undergo photochemical dimerisation through a concerted [2+2] cycloaddition. In 2012, Tedaldi et al. reported that functionalised thiomaleimides, formed through a sequential addition–elimination reaction of thiols with bromomaleimides, were also able to undergo [2+2] cycloaddition under irradiation.^[100]^ As predicted by frontier molecular orbital theory, *exo* head‐to‐head products are preferentially formed. The redshifted absorbance of thiomaleimides relative to the parent maleimide is advantageous, enabling photoactivation with lower energy 365 nm light. This reaction was subsequently exploited for photoinduced peptide macrocyclisation, as well as the re‐bridging of native disulfide bonds in an antibody fragment (Figure 16).^[101]^ The utility of this chemistry was demonstrated by generating an analogue of the therapeutic cyclic peptide octreotide, a synthetic mimic of somatostatin. A key disulfide bridge is essential to the biological activity of octreotide, and the authors showed that linear di‐thiomaleimide peptide **45** indeed showed very low biological activity. Upon irradiation at 365 nm to generate cyclised product **46**, a partial recovery of activity was observed, albeit at levels <10 % of disulfide‐bridged octreotide. Interestingly, it was noted that irradiation led to the formation of four major products of identical mass. This was proposed to be due to the formation of different diastereo‐ and regioisomers, in stark contrast to the high selectivity observed in the intermolecular reactions reported by Tedaldi et al.^[100]^ This serves as another indication that steric restrictions imparted by a peptide substrate can significantly influence regioselectivity.

## Photoinduced Electron Transfer in Peptide Macrocyclisation

### Introduction

Photochemical macrocyclisation can be initiated by quenching of a photoexcited‐state chromophore through electron transfer (ET). This reaction manifold is distinct from the quenching mechanisms discussed above. Photoinduced electron transfer (PET) processes can generate radical ions from neutral starting materials, or neutral radicals from charged precursors. Reaction efficiency is governed by the propensity of these secondary reactive intermediates to undergo macrocyclisation, versus deleterious side reactions or competing back electron transfer (BET) to reform the ground‐state starting materials.[^102^, ^103^, ^104^] In this section, we detail the emerging field of PET‐mediated peptide macrocyclisation, from its origins in UV‐driven intramolecular PET, to applications of intermolecular, visible‐light driven photoredox catalysis, a rapidly developing strategy that is impacting many areas of organic and biomolecular synthesis.

### Intramolecular PET‐initiated peptide macrocyclisation

**Figure 17:**
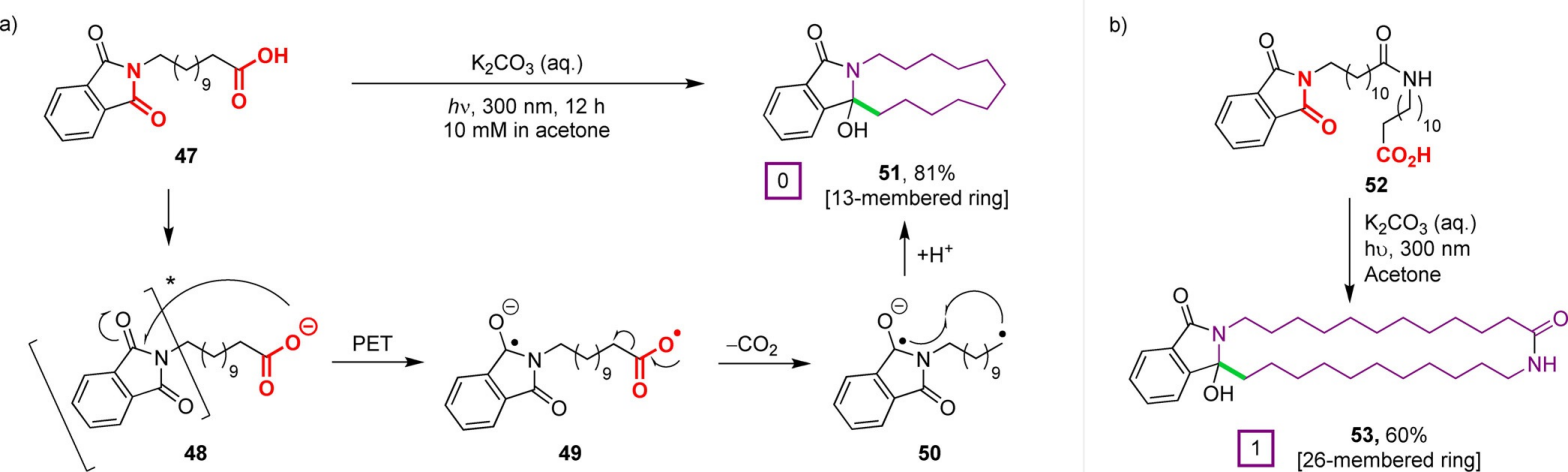
First example of a PET‐initiated macrocyclisation by Griesbeck and co‐workers. a) Mechanism of cyclisation. b) Cyclisation of an amide‐containing substrate.^[106]^

Intramolecular PET‐initiated macrocyclisations on peptide substrates have exclusively exploited ET from donor functional groups to photoexcited phthalimides. Phthalimides are easily incorporated at the N‐termini of peptide chains during solid‐phase peptide synthesis, and upon photoexcitation become highly oxidising (*E* ^S1^=+2.1 V, *E* ^T1^=+1.6 V).^[105]^ Griesbeck and co‐workers performed important early work demonstrating the intramolecular cyclisation of *N*‐phthaloyl ω‐amino acids under UV irradiation.^[106]^ Substrate **47** is prototypical of this approach—on photoexcitation to generate **48**, the phthalimide chromophore underwent PET with the terminal carboxylate, generating a ketyl radical anion on the phthalimide, and a carboxyl radical at the end of the chain, **49** (Figure 17 a). Rapid decarboxylation to form a primary alkyl radical **50** was followed by subsequent biradical intersystem crossing (a triplet state to singlet state transition via the spin flip of an electron) and ensuing cyclisation through radical–radical coupling, to give macrocyclic amidol **51** in 81 % yield. The authors crucially also illustrated the compatibility of this chemistry with substrates containing amide bonds (**52**) in the formation of 26‐membered ring compound **53** (Figure 17 b).

**Figure 18:**
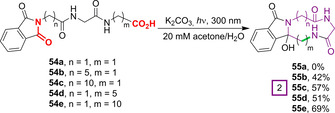
PET‐initiated macrocyclisation of phthalimide‐containing peptide substrates under UV irradiation.[^107^, ^108^]

The first true PET‐mediated PSP macrocyclisations were reported by Griesbeck et al. in 2002 (Figure 18).[^107^, ^108^] Under analogous conditions to those described above, tripeptides **54**, composed of diglycine and a long‐chain unnatural amino acid at either the C or N‐termini, afforded 13‐ or 18‐membered macrocycles **55** in synthetically useful yields. Interestingly, the triglycine derivative **54 a** successfully underwent decarboxylation but then failed to cyclise, instead affording solely the quenched linear *N*‐methyl product in 28 % yield. This behaviour was attributed to hydrogen bonding between the amide proximal to the N‐terminus and the phthalimide unit, which was seemingly disrupted in substrates bearing long‐chain residues, thus enabling cyclisation. By exchanging the central glycine for its *N*‐methylated analogue sarcosine (Sar), this hydrogen‐bonding contribution could be removed and a Gly‐Sar‐Gly tripeptide underwent cyclisation to afford a nine‐membered ring in 35 % yield.

**Figure 19:**
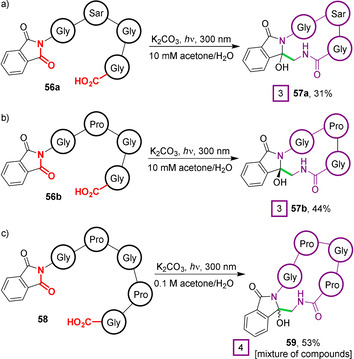
PET‐induced macrocyclisation of phthalimide‐containing peptides composed of proteinogenic amino acids.[^107^, ^108^]

The approach of employing substrates with N‐alkylated proximal amides was subsequently extended to the first example of PET macrocyclisation of a peptide composed solely of proteinogenic amino acids (Figure 19). Cyclisation of the tetrapeptides (Pht)Gly‐Sar‐Gly‐Gly **56 a** and (Pht)Gly‐Pro‐Gly‐Gly **56 b** (Figure 19 a,b) produced 12‐membered ring compounds **57 a** and **57 b**, respectively. Interestingly, **57 b** was isolated as a single diastereoisomer, assigned through a combination of ^1^H NMR analysis and analogy to previously reported benzodiazepines.^[109]^ Finally, this methodology was applied to the macrocyclisation of pentapeptide **58** (Figure 19 c). Proline residues played a vital dual role in enabling cyclisation of this substrate, by both removing the deactivating hydrogen bond at the second residue, and by introducing a hairpin turn that facilitated cyclisation by enhancing the proximity of the N‐ and C‐termini. Unfortunately, however, the presence of prolyl amide bond rotamers and the formation of diastereomers at the amidol position led to the isolation of cyclic tetramer **59** as a complex mixture of species.

Interestingly, in 2003, Yoon, Mariano and co‐workers reported a similar example of PET‐mediated decarboxylative macrocyclisation on an all‐glycine [(Pht)Gly‐Gly‐Gly‐Gly] substrate.^[110]^ Although the proximal amide was not N‐alkylated, and therefore substrate cyclisation might not have been expected on the back of Griesbeck's previous observations, a cyclic trimer was generated in 41 % yield. However, the product was observed to be unstable, with complete decomposition being observed by ^1^H NMR spectroscopy over the period of 1 day. This behaviour was attributed to a deleterious amidol→amido ketone→intermolecular amidol pathway, ultimately forming insoluble oligomers. This process was successfully suppressed through the use of N‐alkylated tertiary amide substrates (see below).

**Figure 20:**
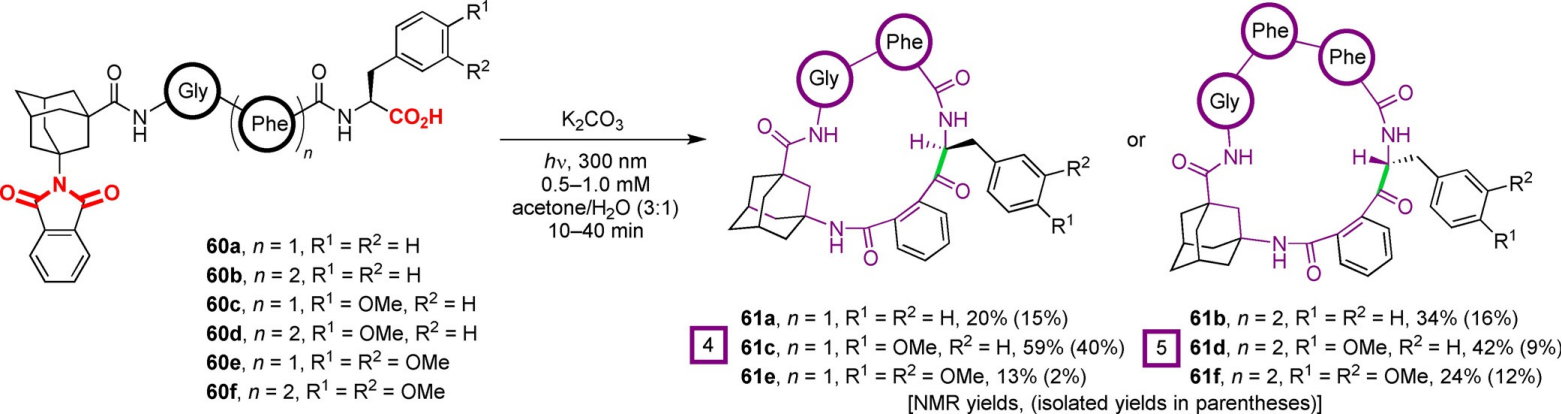
PET‐initiated cyclisation of *N*‐adamantyl phthalimides to C‐terminal phenylalanine derivatives. The stereochemistry of the resultant products was dictated by ring size.^[111]^

Vazdar, Basarić, and co‐workers subsequently applied PET‐mediated decarboxylative cyclisation to tetra‐ and pentapeptides **60** bearing N‐terminal adamantyl phthalimides and C‐terminal phenylalanines, or methoxylated analogues thereof (Figure 20).^[111]^ Products **61** were found to reside as open chain amides, rather than the amidol structures observed in previous reports, and hence are fully peptidic albeit containing an unusual, and potentially metabolically vulnerable, phenyl ketone unit.^[112]^ Interestingly, the macrocycles were formed as single diastereomers (with the exception of **61 d**), with the newly formed chiral centre found to be inverted between 17‐ or 20‐membered ring sizes. The stereochemistry, assigned through a combination of NOESY spectra and molecular dynamics simulations, was determined to result from the conformation of the linear peptides, which controlled the facial approach of the radical species during cyclisation. Incorporation of additional Phe residues in **60 b**,**d**,**f** induced turns in the linear chains, and hence an opposite sense of approach. Possible epimerisation post‐cyclisation was excluded through both computational studies and chemical evaluation through deuterium‐labelling. Substrate‐induced conformational control of a similar biradical macrocyclisation to form an 11‐membered ring product was previously observed by the authors in a separate study. In this instance, however, a single stereogenic centre in the precursor orchestrated the cyclisation with complete stereofidelity through a chiral memory effect.^[113]^

**Figure 21:**
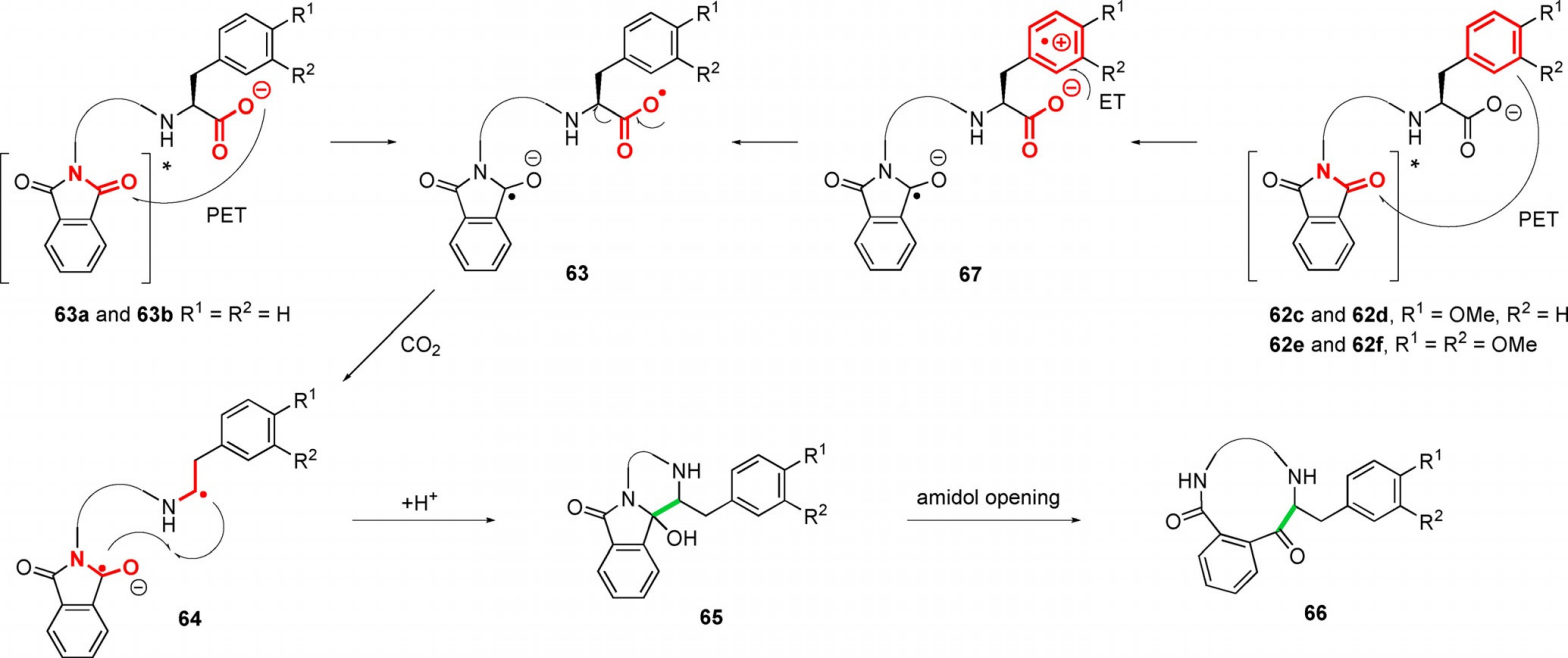
Mechanism of PET‐initiated macrocyclisation of *N*‐adamantyl phthalimides to C‐terminal phenylalanine derivatives. Phenylalanine‐based substrates undergo an analogous PET process to that outlined in Figure 17. In contrast, mono‐ and di‐methoxy‐substituted phenylalanines undergo an intermediate generation of an aryl radical cation.^[111]^

Mechanistically, the macrocyclisation of C‐terminal Phe substrates **60 a** and **60 b** was proposed to proceed in a similar manner to that outlined in Figure 17 a. Direct PET from the carboxylate to the photoexcited‐state phthalimide **62** is followed by decarboxylation (**63** to **64**), triplet to singlet ISC (intersystem crossing), cyclisation (**64** to **65**) and a final amidol to amido ketone ring expansion (**65** to **66**; Figure 21). However, a mechanistic divergence was suggested for the C‐terminal Phe(OMe) and Phe(OMe)_2_ substrates **60 c‐f**, with a more facile initial PET from the electron‐rich arenes to the photoexcited‐state phthalimide taking place, generating aryl radical cations of the type **67**. Subsequent ET from the carboxylate to the aryl group would generate carboxyl radicals **63**, which can then follow the established reactivity pattern. ET from the carboxylate to the more stable dimethoxy radical cations in **67 e** and **67 f** is slower than to the less stable monomethoxy variants **67 c** and **67 d**, reflecting the differences in redox potentials between the two substrates and resulting in lower conversions to the cyclised products **61 e** and **61 f**. Hence, through a balance of increased rates of initial PET from the arene to the photoexcited‐state phthalimide **62**, and a less stable radical cation intermediate **67** promoting rapid ET from the carboxylate, the highest yields were observed for the monomethoxy substates **60 c** and **60 d**. Generally, the isolated yields of the cyclic peptides **61 a**–**d** generated by this methodology are rather low, seemingly reflecting difficulties in purification, which may call into question the synthetic utility (at least at scale) of a study that teaches us much about the nature of these cyclisations.

**Figure 22:**
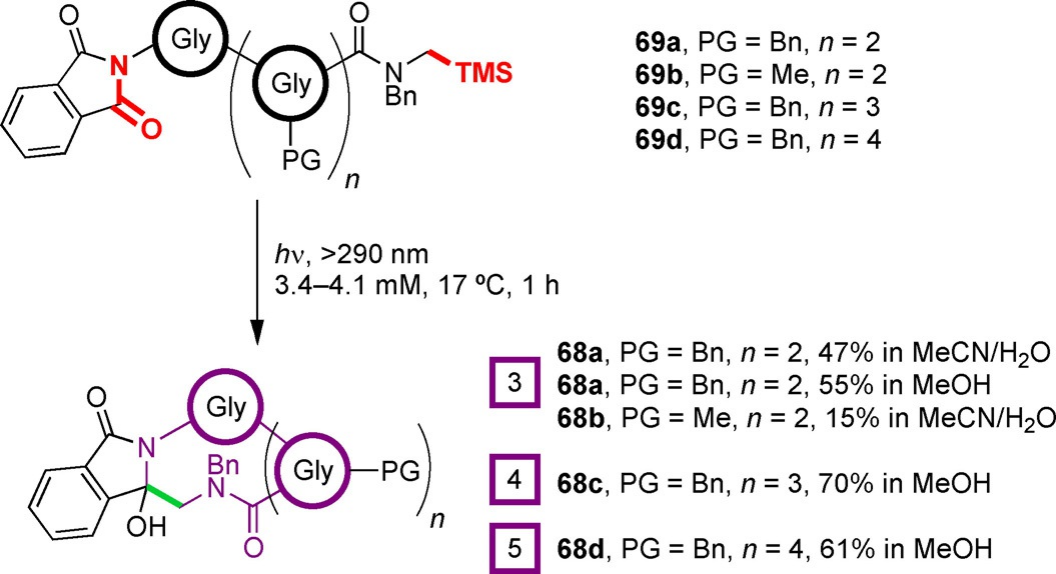
Peptide macrocyclisation via PET from C‐terminal *N*‐trimethylsilylmethyl amides to an excited‐state phthalimide.[^110^, ^114^]

Yoon, Mariano and co‐workers introduced C‐terminal *N*‐trimethylsilylmethyl amides as alternative precursors for PET‐mediated peptide macrocyclisation in 2003.[^110^, ^114^] All‐glycine tri‐, tetra‐ and pentacyclic peptides **68** were prepared in high yields upon UV irradiation of the *N*‐trimethylsilylmethyl amides **69** in either acetonitrile/water mixtures or methanol (Figure 22). Protection of the peptide backbone as *N*‐alkyl tertiary amides was required for the stability of the products.

The authors suggest a mechanism of initial PET between the phthalimide and a proximal amide donor group, followed by amide cation radical migration (or hole transfer) along the peptide backbone to the *N*‐trimethylsilylmethyl amide. Upon desilylation, triplet to singlet ISC and radical–radical cyclisation can then occur.[^110^, ^114^, ^115^] This radical migration mechanism is supported by studies conducted by the authors on polyether‐ and polymethylene‐linked systems, with macrocyclisation yields being lower without electron‐donor atoms in the linker chain. Furthermore, in competition experiments on substrates containing branched chains, and hence two potential sites for reactivity, cyclisation of polyether chains along which radical migration could take place was found to be favoured over hydrocarbon chain cyclisation.^[115]^ Desilylation of *N*‐trimethylsilylmethyl amide radical cations is a rapid process, comparable in rate to the analogous decarboxylation and significantly faster than α‐deprotonation of the intermediate amide radical cation.[^116^, ^117^] Hence, cation radical migration is competitive with BET and α‐deprotonation and therefore yields are largely independent of chain length. It is suggested that electrostatic preorganisation of the phthalimide radical anion/*N*‐trimethylsilylmethyl amide radical cation pair helps to overcome some of the entropic barrier to cyclisation, lowering this energetic cost.

Collectively, these reports demonstrate that PET‐initiated macrocyclisation using excited‐state phthalimide chemistry is potentially a simple and effective strategy to prepare peptide macrocycles. Synthetically useful yields and the possibility to control stereochemistry with substrate conformation are particular advantages of this approach. However, the use of UV light in combination with intramolecular single‐electron transfer (SET) limits the composition of the precursor peptides to a subset of amino acids that do not significantly absorb light at the same wavelengths as phthalimide, and which bear sidechains with oxidation potentials higher than that of the carboxylate donor at the C‐terminus. This greatly limits the versatility and likely precludes the macrocyclisation of peptides containing Asp, Cys, Glu, Met, Sec, Ser, Thr, Trp or Tyr residues, or His and Lys in their free‐base forms. This can be seen in the relatively limited diversity of peptide substrates that have been used. Yield‐limiting hydrogen bonding, substrate instability, high dilution conditions and a requirement for peptide N‐terminal pre‐functionalisation (along with C‐terminal for *N*‐trimethylsilylmethyl amides) are also factors that move this strategy away from being an ideal photochemical strategy for peptide macrocyclisation.

### Peptide macrocyclisation under photoredox catalysis

**Figure 23:**
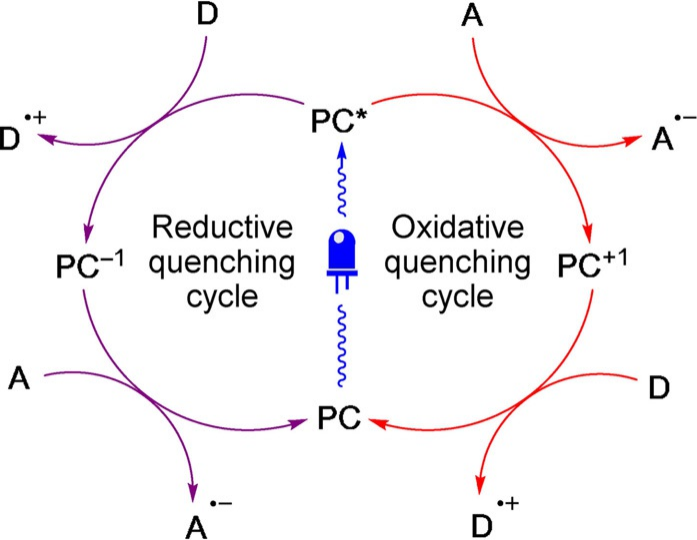
Possible catalytic quenching cycles of a photoexcited photoredox catalyst PC*.

Photoredox catalysis has seen rapid growth in organic synthesis in the last decade, both in terms of application and capability.^[118]^ PET with a photoexcited photoredox catalyst PC* under this manifold can take one of two possible courses (Figure 23): i) an oxidative quenching cycle whereby catalyst PC* transfers an electron to an acceptor species **A**, itself being oxidised to a form **PC^+1^**, which can then accept an electron from a donor **D** to return to the ground‐state **PC**; or alternatively, ii) a reductive quenching cycle proceeding by reduction of the photoexcited‐state catalyst (PC*→**PC^−1^**) by donor **D**, followed by oxidation back to the ground‐state **PC** through ET to acceptor **A**.^[119]^ Acceptor and donor molecules can be reagents, substrates or intermediates generated during the reaction. Whether PC* quenches oxidatively or reductively depends on the best match of the redox potentials of the excited‐state catalyst relative to the species present in the reaction. Various catalytic structures can be exploited, including transition metal polypyridyl complexes,[^119^, ^120^] lanthanide ions,^[121]^ organic compounds,^[122]^ bulk semiconductors[^123^, ^124^] or metal–organic frameworks.^[125]^

When applied to PET‐initiated PSP macrocyclisation, photoredox catalysis offers several potential advantages over a direct intramolecular PET approach. Separating the chromophore from the substrate removes the necessity to pre‐functionalise the PSP with a potentially disruptive unnatural motif. It also gives greater flexibility over chromophore structure, allowing the use of catalysts that absorb lower energy visible light to which all natural amino acids are transparent. This makes photoredox catalysis more compatible with biological species that may suffer damage under UV irradiation.[^53^, ^126^, ^127^, ^128^] Furthermore, with indirect (and therefore asynchronous) ET from the donor to the acceptor through the intermediary of the photocatalyst, more complex and varied chemistries are accessible.[^129^, ^130^, ^131^]

**Figure 24:**
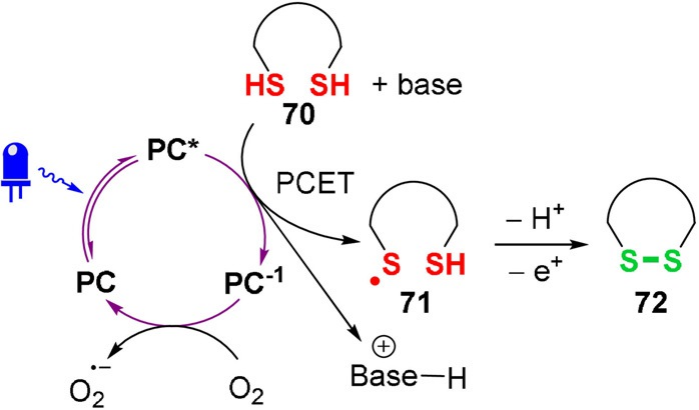
Mechanism of photoredox formation of disulfide bonds via a reductive quenching cycle.

The first reports of peptide macrocyclisation under photoredox catalysis came from the Noël group, as part of their development of photocatalysed thiol oxidations to form disulfides.[^132^, ^133^] The proposed mechanism for this photoredox catalysed transformation commences with photoexcitation of the catalyst with white LEDs, followed by a reductive quenching event through proton‐coupled electron transfer (PCET) from a thiol **70** to PC*, a proposal supported by the observation of increased yields in the presence of base (Figure 24).[^132^, ^133^] The resultant thiyl radical **71** then undergoes disulfide bond formation to form **72**, with the formal loss of a hydrogen atom. This is suggested to occur by sequential thiol deprotonation, thiyl radical addition to the thiolate and single‐electron oxidation of the disulfide radical anion (potentially by superoxide radical anions).[^61^, ^132^] Closure of the catalytic cycle occurs through ET from the reduced form of the photocatalyst **PC^−1^** to molecular oxygen, which acts as the terminal oxidant for this net‐oxidative process. An alternative mechanism where thiyl radical formation occurs through ET to singlet oxygen (^1^O_2_, produced through catalyst‐mediated photosensitisation of ground‐state ^3^O_2_) was discounted owing to the observation of low yields with Ru and Ir photocatalysts, which are known to be capable of generating ^1^O_2_.^[132]^

**Figure 25:**
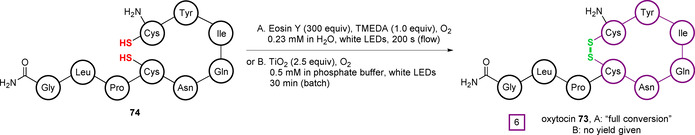
Photoredox‐mediated peptide macrocyclisation to form a disulfide linkage, catalysed by A) eosin Y^[132]^ and B) TiO_2_.^[133]^

This technology was applied to the synthesis of the cyclic peptide hormone oxytocin (**73**, Figure 25). Irradiation of the organic photoredox catalyst eosin Y under an oxygen gas flow in a continuous‐flow photoreactor (200 s residence time) offered a significant rate acceleration over batch conditions, owing to better light penetration and improved oxygen mixing, which helped suppress side reactions (Figure 25, conditions A).^[132]^ Full conversion of linear precursor **74** was observed, with the formation of oxytocin **73** being accompanied with intermolecular disulfide peptide dimers in varying oxidised states, the formation of which was minimised by running the process at higher dilutions. The same group subsequently reported the use of the semiconductor photocatalyst TiO_2_ in batch, to mediate the cyclisation of oxytocin **73** (Figure 25, conditions B). This heterogeneous photocatalyst is attractive as the authors demonstrated that it could be removed by simple filtration or centrifugation, and reused up to ten times with no drop in yield. Although product formation was monitored by LC‐MS, no further isolation or purification was performed and hence the question of synthetic viability remains unanswered in full. However, as a mild method (room temperature, neutral buffer, visible light) employing an easily separated and reusable catalyst, to synthesise naturally occurring disulfide‐bridged macrocycles directly from native peptides bearing two cysteines, this approach shows much promise and merits further investigation.

**Figure 26:**
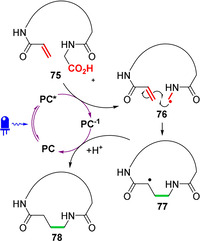
Mechanism of photoredox‐catalysed macrocyclisation via the Giese reaction.

In 2014, MacMillan and co‐workers published an influential paper outlining the Giese reaction (conjugate addition) of small molecule alkyl radicals, generated through the decarboxylation of carboxylic acids under visible‐light photoredox catalysis, to electron‐deficient alkenes.^[134]^ Notably, this chemistry was shown to be well‐suited for radical generation from both *N*‐carbamoyl α‐amino acids and dipeptides. This strategy was subsequently extended to peptide macrocyclisation by the same group in 2017, by incorporating a Michael acceptor radical trap at the peptide N‐terminus (e.g., acrylamide **75**, Figure 26).^[135]^ Mechanistically, PET from the carboxylate form of the C‐terminal acid to the photoexcited catalyst PC* generates a carboxyl radical, which rapidly undergoes loss of CO_2_ to produce α‐amido radical **76**. Following an intramolecular Michael addition, α‐carbonyl radical **77** is reduced to an enolate species through ET from **PC^−1^**, closing the photoredox catalytic cycle and affording macrocycle **78** after protonation. The macrocyclic products of the type **78** are fully peptidic, containing an unnatural γ‐amino acid linkage. The non‐canonical γ‐aminobutyric acid linker is likely to be insensitive to the action of proteases, providing products with increased stability under physiological conditions.

**Figure 27:**
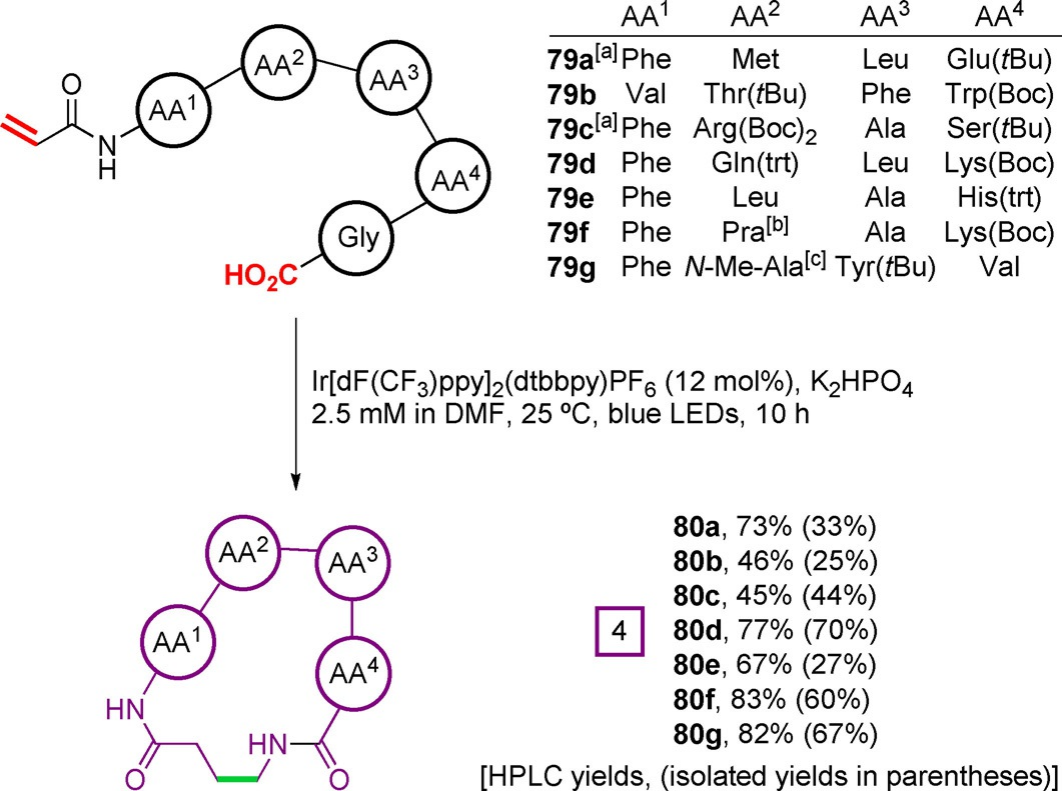
First example of a photoredox‐mediated Giese reaction for peptide macrocyclisation. [a] 10 mol % 2,4,6‐triisopropylthiophenol added. [b] Pra=propargylglycine. [c] *N*‐Me‐Ala=*N*‐methyl alanine.^[135]^

When a series of *N*‐acryloyl pentapeptides with a C‐terminal glycine residue, **79 a**–**g**, were irradiated with a blue LED in the presence of the oxidising iridium photocatalyst Ir[dF(CF_3_)ppy]_2_(dtbbpy)PF_6_, the corresponding macrocyclic peptides **80 a**–**g** were produced efficiently, although isolated yields varied, which is a common observation in this field (Figure 27). The methodology was applied to peptides containing a broad range of amino acids, including non‐canonical propargylglycine (Pra) and *N*‐methyl alanine (*N*‐Me‐Ala). Notably, for examples where there were amino acids bearing polar sidechain, protecting groups were utilised to suppress undesired nucleophilic and/or redox reactions. This lack of functional group tolerance may limit the widespread applicability of this chemistry at the present time. For substrates **79 a** and **79 c**, the addition of 10 mol % 2,4,6‐triisopropylthiophenol to the reaction mixture was found to be beneficial to the yield. This effect was attributed to interception of the α‐carbonyl radical of the form **77** (Figure 26) by H atom abstraction from the thiol at a rate competitive with retro‐Michael addition and unwanted intermolecular oligomerisation, negating the need for rate‐limiting reduction by **PC^−1^**, to provide products **80 a** and **80 c**. In these instances, the catalytic cycle is instead closed by ET from **PC^−1^** to the resultant thiyl radical.

**Figure 28:**
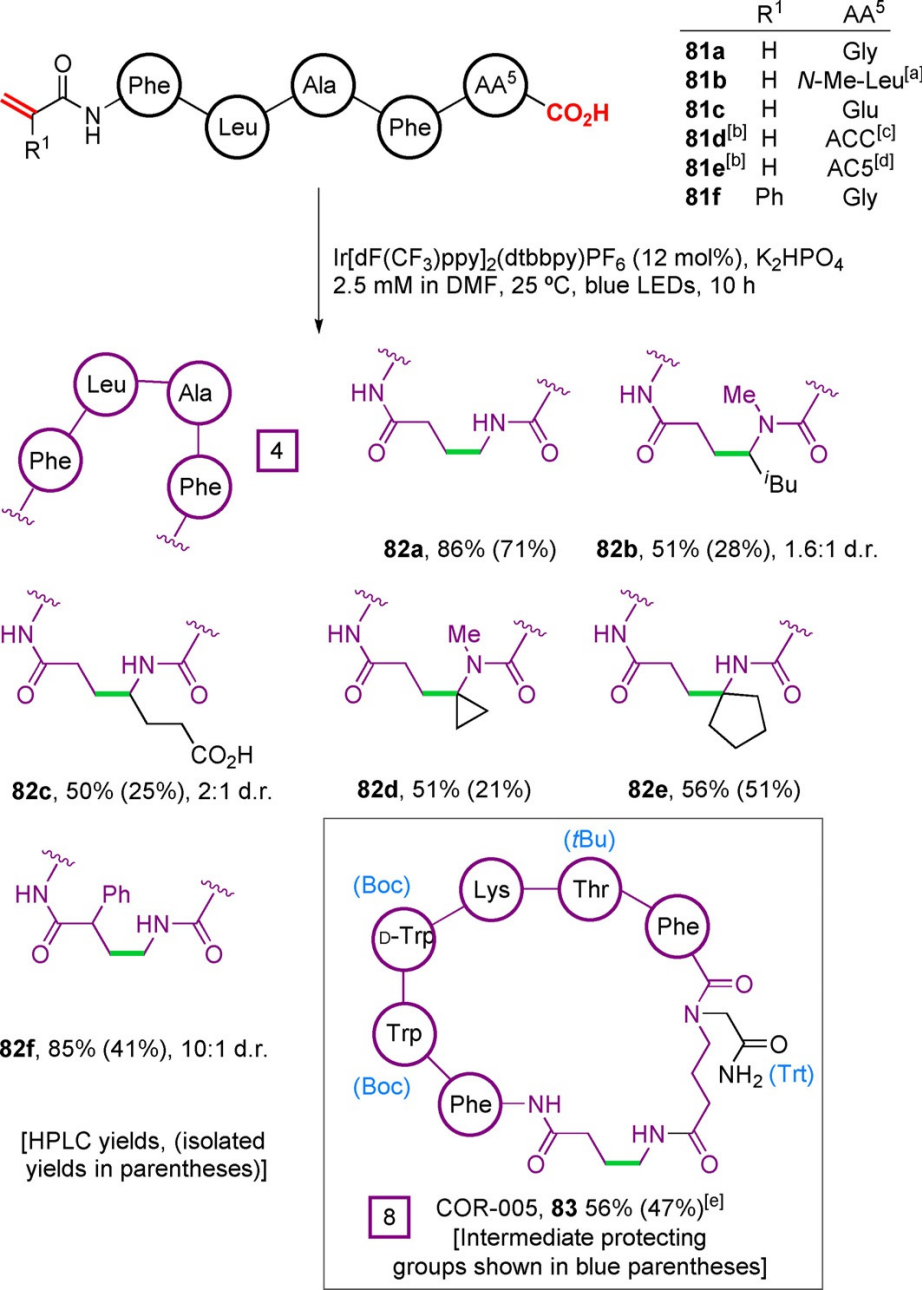
Alternative C‐ and N‐terminal functionalities for the Giese macrocyclisation of peptide substrates. [a] *N*‐Me‐Leu=*N*‐methyl leucine. [b] Reaction performed in DMSO. [c] ACC=1‐Aminocyclopropane‐1‐carboxylic acid. [d] AC5=Cycloleucine. [e] Yield over two steps, following removal of protecting groups with TFA/PhOH/H_2_O/*i*Pr_3_SiH (88:5:5:2) at 0 °C for 2 h.^[135]^

Expanding the scope of the reaction further, the authors explored alternative functionalities at the C‐ and N‐termini. Peptides bearing C‐terminal amino acids with α‐substituents (**81 a**–**f**) were found to be well tolerated and underwent decarboxylative cyclisation, however, yields were reduced relative to terminal glycines (Figure 28). This may be attributable to increased rates of retro‐Michael addition resulting from the increased stability of the substituted α‐amido radicals generated. The formation of diastereomeric product mixtures was found to occur with little control (**81 b** and **81 c**). Notably, substrate **81 c** with a C‐terminal glutamic acid underwent chemoselective α‐amino decarboxylation on account of the lower p*K*_a_ and oxidation potential of the C‐terminus relative to the γ‐carboxylic acid.^[136]^ Although not demonstrated, analogous selectivity over aspartic acid sidechains would also be expected for the same reasons. Unnatural di‐α‐substituted cyclic amino acids at the peptide C‐terminus were able to form unusual spirocyclic peptide macrocycles (**82 d** and **82 e**). At the N‐terminus, only a single functionalised acrylamide motif was investigated, with an electronically activating phenyl group at the α‐position leading to the formation of macrocycle **82 f** in high yield and with good diastereoselectivity. The methodology performed well when applied to the synthesis of larger ring sizes, with remarkably little yield variation observed across the preparation of 8‐, 10‐ and 15‐membered cyclic peptides. Importantly, the authors also demonstrated the straightforward post‐cyclisation removal of acid‐labile protecting groups, leading to the generation of the somatostatin receptor agonist peptide COR‐005 (**83** in 47 % isolated yield over two steps).

### Peptide macrocyclisation under dual photoredox and transition‐metal catalysis

Advances in the field of photoredox catalysis have been mirrored in their application in PSP macrocyclisation. At the cutting edge of this area is the merger of photoredox and transition‐metal catalysis. This extremely powerful combination opens up unprecedented chemical transformations that are well‐suited to applications in complex settings on account of the mild reaction conditions.[^131^, ^137^, ^138^] Key to the success of this approach is the activation of transition‐metal complexes by a photocatalyst, either through redox modulation or energy transfer pathways, triggering mechanistic steps that would not be operative under transition‐metal catalysis alone.[^139^, ^140^, ^141^, ^142^, ^143^, ^144^]

The Sciammetta group disclosed a methodology for the etherification of peptidic alcohols with aryl bromides by C(sp^2^)−O cross‐coupling under dual photoredox and nickel catalysis.^[145]^ In addition to intermolecular ether formation, the reaction conditions were also shown to be amenable to macrocyclisation of a series of N‐terminal bromobenzamides, via aryl etherification of C‐terminal serine derivatives. The authors proposed this methodology to target underutilised ether macrocyclic linkages, which they anticipated would overcome several of the shortcomings at times exhibited by other macrocycle chemistries, such as proteolytic instability (e.g., thioether linkages), and the poor cell‐permeability of hydrogen bond donor containing linkages (e.g., those with an N−H bond).

**Figure 29:**
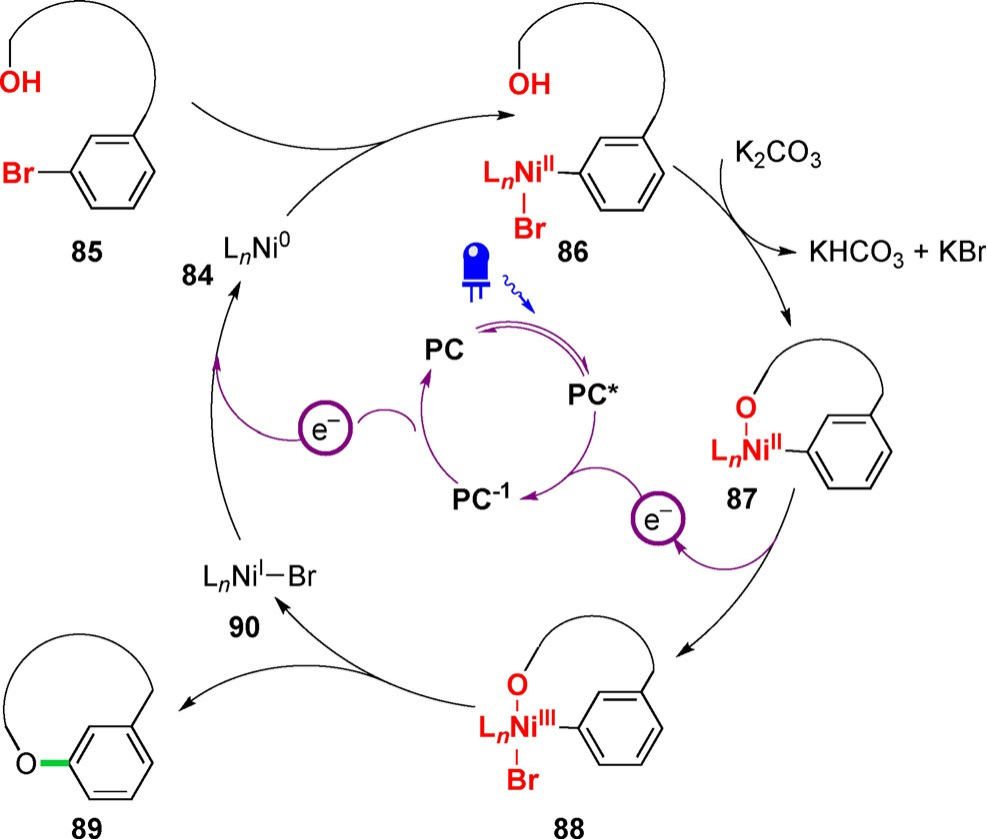
Mechanism of dual photoredox–nickel catalysis for the macrocyclic etherification of alcohols with aryl bromides.

The proposed mechanism of this reaction was analogous to that previously reported by MacMillan and co‐workers for the dual photoredox–nickel catalysed arylation of small‐molecule alcohols (Figure 29).^[139]^ Following the oxidative addition of a Ni^0^ complex **84** into an aryl bromide **85**, the resultant aryl‐Ni^II^ species **86** can undergo ligand exchange to form cyclic Ni^II^ alkoxide **87**. Single‐electron oxidation of complex **87** by ET to the excited‐state photocatalyst PC* generates a key Ni^III^ aryl alkoxide **88**, which, unlike Ni^II^ aryl alkoxide complex **87**, is unstable with respect to reductive elimination and the catalytic cycle therefore generates peptide macrocycle **89** and the Ni^I^ species **90**. Finally, ET to **90** from **PC^−1^** then regenerates Ni^0^ species **84** and simultaneously closes both catalytic cycles.

**Figure 30:**
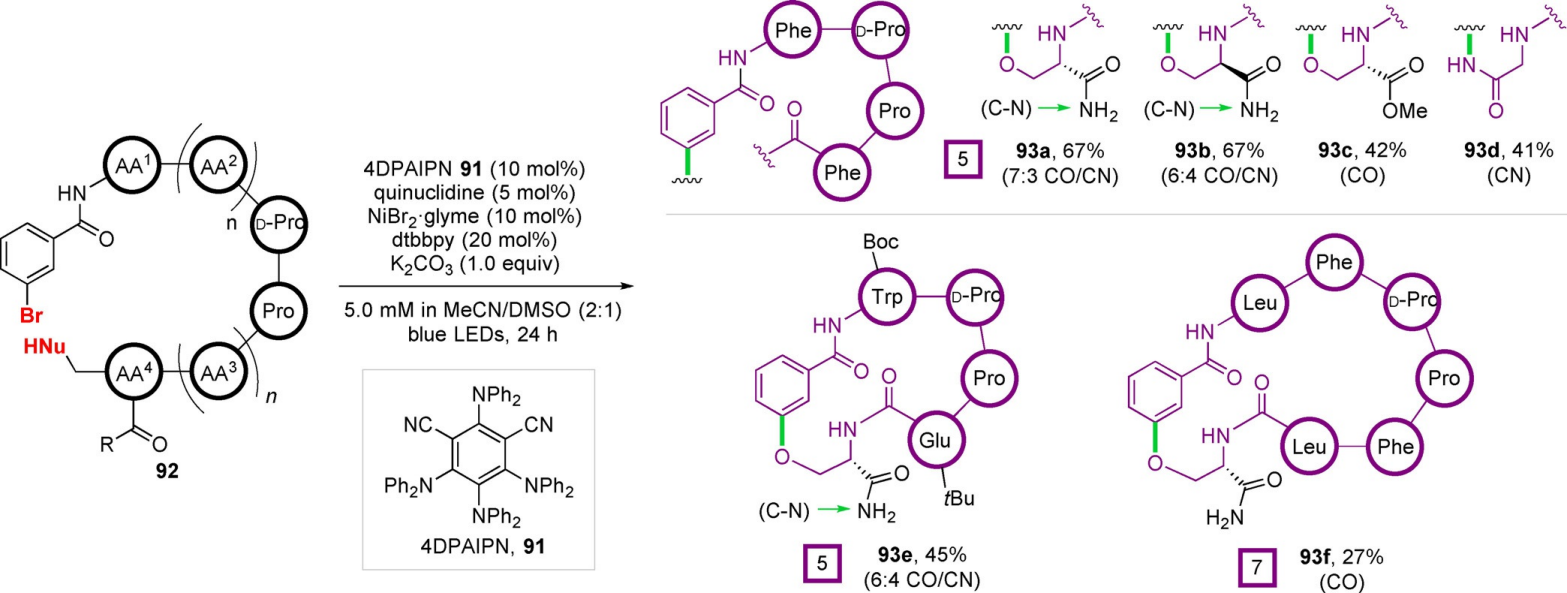
Dual photoredox–nickel catalysis for peptide macrocyclisation.^[145]^

Using the isophthalonitrile‐based organic photocatalyst 4DPAIPN **91** and a NiBr_2_ **⋅**glyme/dtbbpy transition‐metal catalyst system, in concert with quinuclidine as a yield boosting additive (an affect attributed to its capacity to act as an electron donor or shuttle), the authors were able to induce macrocyclisation of a series of N‐terminal *m*‐bromobenzamide modified peptides **92 a**–**f** (Figure 30). All substrates contained a β‐hairpin inducing d‐Pro‐l‐Pro subunit to preorganise the linear peptide for cyclisation via rigidifying hydrogen bonds, leading to the formation of cyclised peptides **93**. In the absence of this dipeptide, only trace yields of macrocycle formation were detected by UPLC‐MS. The importance of these hydrogen bonds was supported by in silico conformational sampling by a distance geometry approach. For pentapeptide substrates with C‐terminal serinamides (**92 a**,**b**,**e**), competing O‐ and N‐arylation was observed to provide ether‐ and amide‐linked macrocycles, which were separable by HPLC. This competitive cyclisation favoured the ether over amide linkages by ratios of 6:4–7:3 (see Figure 30). For substrates with a C‐terminal ester (**92 c**) or glycinamide (**92 d**) residues, presenting a single nucleophilic position, macrocyclisation proceeded exclusively through etherification or amidation, respectively. Variation of the peptide sequence to incorporate residues bearing protected polar functionality (**92 e**) or increasing the ring size (**92 f**) was also successful. Interestingly, macrocyclisation in the presence of a free carboxylic acid was not demonstrated, potentially because of competing lactonisation. This transformation has been demonstrated in an intermolecular sense by an energy‐transfer mechanism under similar reaction conditions.^[140]^ Notably, the requirement for the protection of polar residues, and more importantly the presence of a turn‐enforcing d‐Pro‐l‐Pro subunit to achieve good cyclisation efficiency are limitations that need to be addressed for this methodology to achieve broad synthetic applicability in peptide macrocyclisation.

## Conclusion

In this review, we have detailed the photochemical strategies that have been exploited to induce PSP macrocyclisation. These methods benefit from mild reaction conditions, the ability to selectively introduce energy and the sheer variety of reaction modes available. Developments in this field have mirrored the recent renewed interest in photochemistry, and this looks set to continue as macrocyclic PSPs become increasingly prevalent in the drug discovery pipeline. The rapid expansion of new photochemical strategies in organic synthesis provides a rich source of potential new reactions to control cyclisation. Indeed, there remains significant scope for innovation in the area, with a pressing need to both evolve existing strategies for cyclisation, and develop new ones.

In particular, we see an urgent need for new reactions that can overcome limitations in amino acid tolerance. It is notable that in many of the examples presented within this review, the linear peptide substrates are composed of a very simple set of afunctional amino acids with hydrocarbon sidechains. The resultant methodologies, while synthetically interesting, provide little scope to generate macrocyclic peptides with useful biological properties. A lack of functional group tolerance is also highlighted by the common reliance on fully protected peptides as substrates. Exceptions, such as thiol‐“ene” cyclisations, must proceed with exquisite chemoselectivity to avoid side reactions with the myriad of reactive functionalities found within unprotected, canonical amino acids. Recent developments in photoredox catalysis, enabling site‐selective modification of even complex proteins, are therefore particularly exciting, although it should be noted that even then the presence of a subset of amino acids must be avoided.[^136^, ^146^] New reactions that can increase this selectivity still further would be invaluable to the bioconjugate community, and would provide an important and generalisable tool for peptide macrocyclisation.

Moreover, we anticipate increased use of reactions that exploit lower energy visible light sources to induce macrocyclisation, with associated improvements in selectivity. The reliance of many of the reactions described here on high energy UV irradiation is inherently limiting to functional group tolerance.^[147]^ Photoredox catalysis driven by visible light is an important avenue to overcome this limitation, and we anticipate a greater application of this technology to the macrocyclisation of PSPs. Other visible light‐driven photochemical catalysis manifolds, such as energy transfer and photon upconversion, are also well‐positioned for implementation in PSP macrocyclisation. With these innovations the prospect of achieving general strategies for *protein* macrocyclisation will become more realistic, adding to the fairly limited toolkit of photochemical reactions that have been exploited for this ambitious goal to date.[^74^, ^101^]

Finally, the development of photochemical reactions that can deliver unique macrocyclic linkages represents an important future challenge for the community. The diversity of reaction manifolds that are accessible through photochemical approaches is attractive given the importance of even subtle differences in peptide linker structure on biological properties, such as membrane permeability and target binding.^[71]^ New reactions that can be used to modulate the properties of the resultant cyclic peptides would therefore represent valuable tools in the search for novel peptide therapeutics. Important challenges that need to be overcome in this regard include the stereocontrol of newly formed chiral centres, a difficult task in such a functionally dense environment, and a reduced reliance on turn‐inducing residues to aid cyclisation.

We anticipate the benefits of light‐mediated chemistry will be increasingly exploited in the future as the field matures. Exciting opportunities for spatial and temporal control over cyclisation may enable applications in advanced biomedical technologies, diversifying away from the traditional roles of cyclised peptides as therapeutic agents. As the journey towards more ‘ideal’ macrocyclisation techniques that are readily applicable to PSP substrates and that tolerate ever increasing functionality continues, the future for photochemical macrocyclisation methods looks bright.

## Conflict of interest

The authors declare no conflict of interest.

## Biographical Information

*Laetitia Raynal studied Chemistry at the École Nationale Supérieure de Chimie de Montpellier, with a major in Materials Chemistry. During her Master′s, she did an internship at DSM in the Netherlands and a one‐year research placement at the University of Geelong in Australia, graduating with a Diplôme d'ingénieur Chimiste in 2019. The same year, she was awarded a Rosetrees Trust PhD Scholarship with Dr. Chris Spicer at the University of York, developing new approaches to enhance protein signalling*.

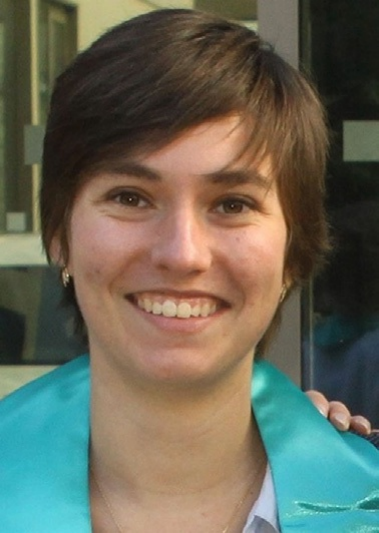

## Biographical Information

*Nicholas C. Rose received his MSci of Chemistry with a Year in Industry in 2019 from the University of Nottingham, following an industrial placement year in 2018 at Merck Chemicals Ltd. in Southampton. In 2019, he was awarded an EPSRC PhD Scholarship with Dr. Chris Spicer at the University of York. His current research interests involve developing novel dynamic biomaterial conjugation systems for tissue engineering*.

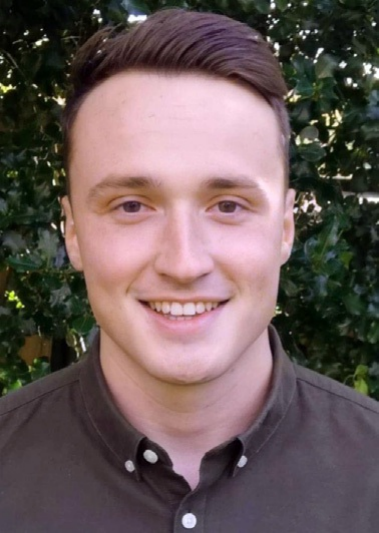

## Biographical Information

*James Donald studied Natural Sciences at the University of Cambridge, and then obtained his PhD at the University of York with Prof. Richard Taylor. Following postdoctoral research at the University of Texas at Austin and the University of Oxford, he joined Prof. David MacMillan's group at Princeton University. On returning to York, he has worked with Prof. Richard Taylor, Prof. Peter O'Brien and Dr. Chris Spicer. His research interests centre on applications of photoredox catalysis in novel bond construction and the synthesis of molecules of medicinal relevance*.

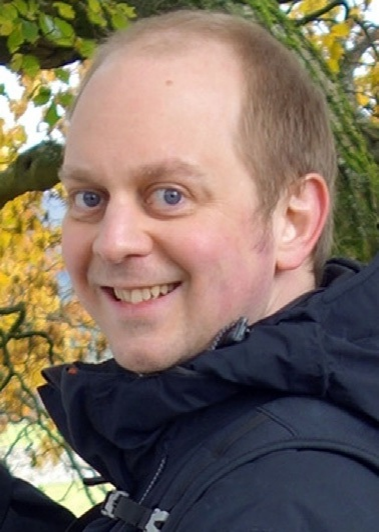

## Biographical Information

*Chris Spicer is a Lecturer in Chemistry at the University of York, where his group is interested in the design and chemical synthesis of novel biomaterials and bioconjugates for tissue repair. Previously, he studied Natural Sciences at the University of Cambridge, before moving to the University of Oxford to undertake a PhD with Prof. Ben Davis. Chris went on to complete postdoctoral research with Prof. Molly Stevens, first at Imperial College London and then the Karolinska Institutet in Stockholm*.